# iRhom2 regulates ERBB signalling to promote KRAS-driven tumour growth of lung cancer cells

**DOI:** 10.1242/jcs.259949

**Published:** 2022-09-08

**Authors:** Boris Sieber, Fangfang Lu, Stephen M. Stribbling, Adam G. Grieve, Anderson J. Ryan, Matthew Freeman

**Affiliations:** 1 Sir William Dunn School of Pathology, University of Oxford, Oxford OX1 3RE, UK; 2 Department of Oncology, University of Oxford, Oxford OX3 7DQ, UK

**Keywords:** KRAS, iRhom2, ERBB, EGFR, ADAM17, A549 cells

## Abstract

Dysregulation of the ERBB/EGFR signalling pathway causes multiple types of cancer. Accordingly, ADAM17, the primary shedding enzyme that releases and activates ERBB ligands, is tightly regulated. It has recently become clear that iRhom proteins, inactive members of the rhomboid-like superfamily, are regulatory cofactors for ADAM17. Here, we show that oncogenic KRAS mutants target the cytoplasmic domain of iRhom2 (also known as RHBDF2) to induce ADAM17-dependent shedding and the release of ERBB ligands. Activation of ERK1/2 by oncogenic KRAS induces the phosphorylation of iRhom2, recruitment of the phospho-binding 14-3-3 proteins, and consequent ADAM17-dependent shedding of ERBB ligands. In addition, cancer-associated mutations in iRhom2 act as sensitisers in this pathway by further increasing KRAS-induced shedding of ERBB ligands. This mechanism is conserved in lung cancer cells, where iRhom activity is required for tumour xenograft growth. In this context, the activity of oncogenic KRAS is modulated by the iRhom2-dependent release of ERBB ligands, thus placing the cytoplasmic domain of iRhom2 as a central component of a positive feedback loop in lung cancer cells.

This article has an associated First Person interview with the first authors of the paper.

## Abstract (precis)

**Summary:** Oncogenic KRAS mutations lead to phosphorylation of iRhom2, driving a tumour-promoting feedback loop of ADAM17-dependent ERBB signalling. Cancer-associated mutations in iRhom2 enhance this signalling.

## INTRODUCTION

The ERBB/EGFR signalling pathway is dysregulated in numerous cancers, especially of the lung, breast and ovary (Sigismund et al., 2018; Wang, 2017). In addition to oncogenic receptor mutations, tumorigenesis can be driven by excess ERBB ligand production (Arteaga and Engelman, 2014). ERBB family ligands are mostly synthesised as type I transmembrane domain proteins, and become active upon proteolytic cleavage and release (shedding) from the plasma membrane. Thus, shedding of ERBB ligands is a primary regulator of signalling that controls pathogenesis as well as cell proliferation, survival and differentiation.

This mode of regulation puts into the spotlight the enzymes responsible for shedding ligands. The metalloprotease ADAM17 is the most widespread sheddase of ERBB ligands, as well as controlling the release of many other growth factors, cytokines and other cell surface proteins (Zunke and Rose-John, 2017). Consistent with its potency, an intricate regulatory mechanism exists to control ADAM17, centred on iRhom1 and iRhom2 (also known as RHBDF1 and RHBDF2, respectively), which are rhomboid-like proteins that act as ADAM17 cofactors (Dulloo et al., 2019). iRhom proteins (iRhoms) are first required for the trafficking of ADAM17 from the endoplasmic reticulum (ER) to the Golgi, where the protease is matured by removal of its inhibitory pro-domain by the pro-protein convertase furin proteins (Adrain et al., 2012; Endres et al., 2003; McIlwain et al., 2012). iRhom2 has subsequently been shown to have an additional role at the plasma membrane – it regulates the activation of ADAM17 to release TNFα, the major type I inflammatory cytokine. This activation of ADAM17 by iRhom2 is triggered by phosphorylation of the cytoplasmic domain of iRhom2 mediated by ERK1 and ERK2 (ERK1/2; also known as MAPK3 and MAPK1, respectively) (Cavadas et al., 2017; Grieve et al., 2017).

Several lines of evidence have implicated iRhoms and ADAM17 in tumorigenesis, especially in lung, breast, cervical, oesophageal and colorectal cancers. iRhom2 and ADAM17 levels increase during cancer progression and correlate with lower survival rates (Agwae et al., 2020; Borrell-Pagès et al., 2003; Mochizuki and Okada, 2007; Walkiewicz et al., 2016; Xu et al., 2020; Zhou et al., 2006, 2014). The most direct link between the iRhom proteins and cancer are mutations in the cytoplasmic domain of iRhom2, which cause a rare familial syndrome, tylosis with oesophageal cancer (TOC), characterised by a very high lifetime risk of developing oesophageal cancer (Blaydon et al., 2012; Ellis et al., 2015; Mokoena et al., 2018; Qu et al., 2019; Saarinen et al., 2012). Increased activity of ADAM17 has been observed for *iRhom2^TOC^* mutations (Brooke et al., 2014; Maney et al., 2015) but, despite this strong genetic link, the precise mechanistic role of iRhoms in oncogenic signalling has been poorly explored.

ADAM17 is better characterised than iRhoms with respect to cancer, although until recently it too has not been the subject of the intense focus commensurate with its regulatory importance. For instance, oncogenic SRC triggers the ADAM17-dependent release of the ERBB ligand TGFα (Maretzky et al., 2011). It has also become clear that ADAM17 is important in cancers mediated by mutations in *KRAS*, which are the most frequent oncogenic mutations in human cancers, particularly in lung, colorectal and pancreatic tumours (Khan et al., 2019). Although oncogenic KRAS has long been considered to be constitutively active, and thus independent of upstream signals, a requirement for ADAM17 and ERBB1/EGFR in KRAS-induced pancreatic cancer has challenged this idea (Ardito et al., 2012; Navas et al., 2012). Indeed, ERBB signalling has now been shown to contribute to lung tumorigenesis by supporting activation of oncogenic KRAS (Kharbanda et al., 2020; Kruspig et al., 2018; Moll et al., 2018). In this context, it is also significant that KRAS-driven tumours express higher levels of ERBB ligands, in particular amphiregulin and TGFα (Ardito et al., 2012; Kruspig et al., 2018). However, as described above, ERBB ligands must be proteolytically shed to be active, and the regulation of shedding in cancer has been largely unknown. A recent advance has been the demonstration of a requirement for ADAM17 in KRAS-induced lung tumorigenesis (Saad et al., 2019). Using non-small-cell lung carcinoma (NSCLC) and patient-derived xenografts, as well as the *Kras^G12D^* mouse model, Saad et al. showed that depletion of ADAM17, or inhibition of its activity, suppressed lung tumour growth. They also found that oncogenic KRAS leads to increased activity of the p38 MAPKs and upregulated shedding of the ADAM17 substrate IL-6R.

Here, we report that iRhoms are essential for the oncogenic release of ERBB ligands mediated by KRAS-G12 mutants. Specifically, KRAS-induced shedding of ERBB ligands is triggered by the phosphorylation of the cytoplasmic domain of iRhom2, which allows the recruitment of the phospho-binding 14-3-3 proteins. Human cancer-associated mutations in the cytoplasmic domain of iRhom2 are sufficient to amplify this pathway, thus further establishing iRhom2 as an important component of oncogenic signalling. The pathological significance of this pathway was validated upon oncogenic KRAS expression in HEK239T cells and in the NSCLC cell line A549, which harbours an endogenous oncogenic *KRAS^G12S^* mutation. Furthermore, loss of iRhom activity completely suppressed KRAS-driven tumour xenograft growth, demonstrating the requirement of iRhoms in a widely used model of lung cancer. Finally, we report that the cytoplasmic domain of iRhom2 is a hub for an ERBB-dependent positive feedback loop that maintains KRAS activity in lung cancer cells. Overall, our results demonstrate that iRhom2 plays a central role in oncogenic KRAS-induced signalling.

## RESULTS

### iRhoms are required for KRAS-driven shedding of ERBB ligands by ADAM17

**Fig. 1.**
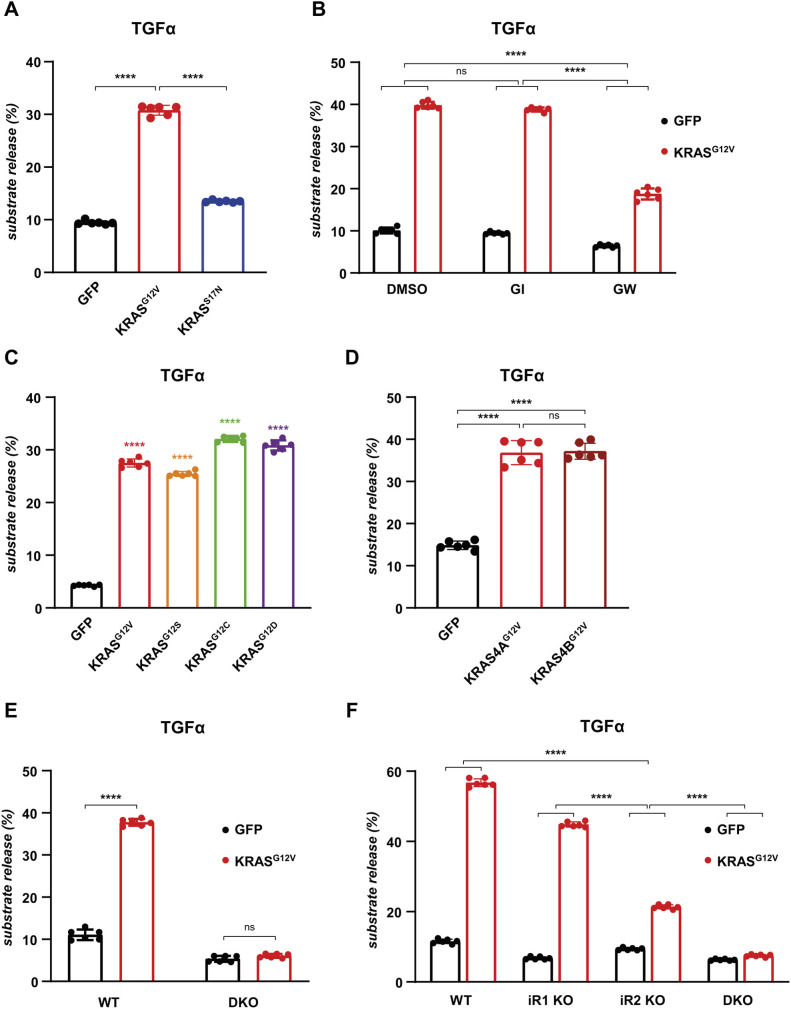
**iRhoms are required for KRAS-driven shedding of the ERBB ligands by ADAM17.** (A–D) HEK293T cells were co-transfected with alkaline phosphatase (AP)-tagged TGFα and GFP or GFP-tagged KRAS constructs. Unless specified otherwise, constructs of KRAS4A were used in all experiments. Overnight medium collection was performed in the presence of 0.5 μM ADAM10 inhibitor (GI), 0.5 μM ADAM10 and ADAM17 inhibitor (GW) or with DMSO. (E,F) Wild-type, iRhom1 KO, iRhom2 KO, or iRhom1/2 double knockout (DKO) HEK293T cells were transiently co-transfected with AP-tagged TGFα and GFP or GFP-tagged KRAS-G12V, followed by overnight medium collection. Substrate release is the level of alkaline phosphatase in the medium divided by the total alkaline phosphatase level. Data are from six biological replicates. Error bars represent s.d. ****P<0.0001; ns, not significant (*P*>0.05) [one-way ANOVA and Tukey multiple comparison test on log transformed (A,C,F) or untransformed (B,D,E) data].

Oncogenic KRAS induces the activation of ADAM17 (Ardito et al., 2012; Saad et al., 2019; Van Schaeybroeck et al., 2011), so we questioned whether iRhoms play a role in this process. First, to establish the effect of oncogenic KRAS in HEK293T cells, we expressed oncogenic KRAS^G12V^. As expected, we observed a significant increase in the release of the ADAM17 substrate TGFα (Fig. 1A) especially compared to the effect of KRAS^S17N^ (Fig. 1A), a mutant with reduced GTPase activity (Farnsworth and Feig, 1991; Feig and Cooper, 1988). Using the inhibitors GI254023X and GW280264X, which respectively inhibit ADAM10, or both ADAM10 and ADAM17 (Hundhausen et al., 2003), we confirmed that TGFα shedding mediated by oncogenic KRAS was dependent on ADAM17 (Fig. 1B), which agrees with the reported ability of oncogenic KRAS to increase ADAM17-dependent shedding (Ardito et al., 2012; Saad et al., 2019; Van Schaeybroeck et al., 2011).

Although KRAS^G12V^ is one of the most well-studied oncogenic forms of KRAS (Muñoz-Maldonado et al., 2019), several other *KRAS^G12X^* mutations are found in human cancers (Hobbs et al., 2016; Timar and Kashofer, 2020). We found that KRAS^G12S^, KRAS^G12C^ and KRAS^G12D^ all caused elevated TGFα release (Fig. 1C). We also demonstrated that both isoforms of KRAS, 4A and 4B, induce shedding of TGFα (Fig. 1D). Overall, these results demonstrate the shared ability of KRAS oncogenic mutants to trigger growth factor release.

Having shown that oncogenic mutations in KRAS induce ADAM17-dependent shedding of TGFα, we next asked whether iRhoms are required for this activity. We found that KRAS-induced shedding of TGFα was completely blocked in HEK293T double-knockout (DKO) cells mutant for both iRhom1 and iRhom2 (Fig. 1E; Fig. S1). In single knockout lines, loss of iRhom1 had little effect, whereas iRhom2 KO showed a strong reduction in TGFα shedding (Fig. 1F), thereby demonstrating that iRhom2 is the primary mediator of KRAS-induced ADAM17-dependent shedding of TGFα.

### KRAS-induced shedding depends on phosphorylation of the cytoplasmic domain of iRhom2 by the Raf/MEK/ERK pathway

**Fig. 2.**
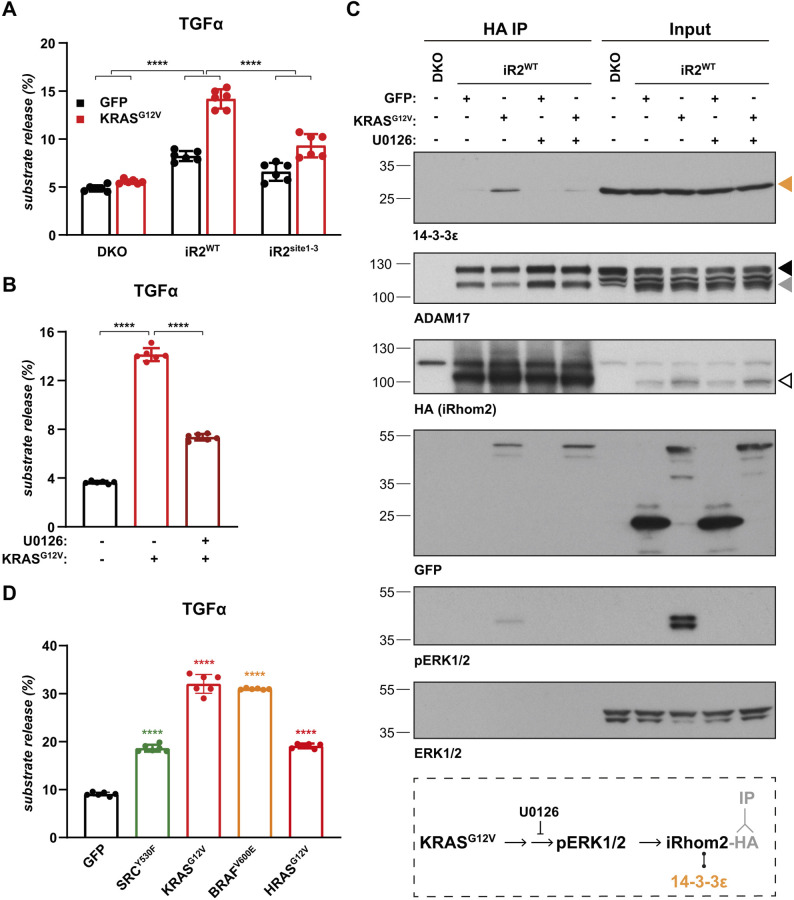
**KRAS-induced shedding depends on the phosphorylation of the cytoplasmic domain of iRhom2 by the Raf/MEK/ERK pathway.** (A) iRhom1/2 DKO HEK293T cells reconstituted with iRhom2^WT^ and iRhom2 lacking the three primary phosphorylation sites (iRhom2^site1-3^) were co-transfected with GFP or GFP-tagged KRAS^G12V^ and alkaline phosphatase (AP)-tagged TGFα, and medium was collected overnight. Error bars represent s.d. ****P<0.0001 for the induction ratio (KRAS^G12V^/GFP) (one-way ANOVA and Tukey multiple comparison test). (B) iRhom1/2 DKO HEK293T reconstituted with iRhom2^WT^ and co-transfected with GFP or GFP-tagged KRAS^G12V^ and AP-tagged TGFα were treated with 10 μM U0126 during 3 h of medium collection. Data are from six biological replicates. Error bars represent s.d. ****P<0.0001 for the log transformed data (one-way ANOVA and Tukey multiple comparison test). (C) HA-based immunoprecipitates and lysates from iRhom1/2 DKO HEK293T cells reconstituted with HA–iRhom2^WT^ and transfected with GFP or GFP-tagged KRAS^G12V^ were immunoblotted for 14-3-3ε, ADAM17, HA and GFP. To assess the contribution of Raf/MEK/ERK cascade, cells were treated with 10 μM U0126 for 2 h and blotted for phosphorylated ERK1/2 (pERK1/2). Orange arrowhead indicates 14-3-3ε, black and grey arrowheads indicate immature proADAM17 and mature ADAM17, respectively, white arrowhead indicates HA-tagged iRhom2^WT^. The experiment was repeated three times. A schematic of the rationale of the experiment is shown below the immunoblot. (D) HEK293T cells were transiently co-transfected with AP-tagged TGFα and GFP or GFP-tagged ERK-activating oncogenes SRC^Y530F^, KRAS^G12V^, BRAF^V600E^, HRAS^G12V^, followed by overnight medium collection. Data are from six biological replicates. Substrate release is the level of released alkaline phosphatase in the medium divided by the total alkaline phosphatase level. Error bars represent s.d. ****P<0.0001 for log transformed data (one-way ANOVA and Tukey multiple comparison test).

To determine whether, as in inflammatory signalling (Cavadas et al., 2017; Grieve et al., 2017), iRhom2 phosphorylation participates in oncogenic ADAM17 signalling, we used a mutant version of iRhom2 (iRhom2^site1-3^) in which the three primary phosphorylation sites are changed to alanine residues (Grieve et al., 2017; Fig. S2A). We found that without iRhom2 phosphorylation at these three main sites, shedding of TGFα was significantly reduced (Fig. 2A). Importantly, this phosphorylation-deficient form of iRhom2 supported ADAM17 maturation as efficiently as wild-type iRhom2 (iRhom2^WT^; Fig. S2B), which aligns with our previous findings that iRhom2 phosphorylation is not needed for ADAM17 maturation (Grieve et al., 2017). These results reveal the role of iRhom2 phosphorylation in oncogenic signalling by KRAS.

In inflammatory signalling, iRhom2 phosphorylation is MAPK dependent (Cavadas et al., 2017; Grieve et al., 2017); it is also well established that oncogenic KRAS mutations act through the Raf/MEK/ERK MAPK pathway (Muñoz-Maldonado et al., 2019; Schubbert et al., 2007). We therefore asked whether the RAS/MAPK cascade also participates in iRhom2-dependent oncogenic signalling. TGFα shedding induced by KRAS^G12V^ was strongly inhibited by treating the cells with U1026 (Fig. 2B), a specific inhibitor of MEK1 and MEK2 (MEK1/2; also known as MAP2K1 and MAP2K2, respectively) (Favata et al., 1998), the kinases upstream of ERK1/2. We also found that oncogenic KRAS triggers the recruitment of 14-3-3 epsilon to iRhom2 and that, consistent with 14-3-3 proteins binding to phosphorylated residues (Fu et al., 2000), this recruitment was inhibited by treatment with U1026 (Fig. 2C; Fig. S2C). 14-3-3 recruitment to iRhom2 was associated with decreased binding between iRhom2 and ADAM17 (Fig. 2C). Although we have not investigated this phenomenon further, it agrees with our previous work on inflammatory signalling (Grieve et al., 2017) and suggests that the activation of ADAM17 by phosphorylated iRhom2 depends on an altered interaction between them. Since the recruitment of 14-3-3 to iRhom2 is sufficient for ADAM17 activation (Cavadas et al., 2017; Grieve et al., 2017), these results demonstrate that KRAS-induced shedding of ERBB ligands is mediated by ERK1/2-dependent phosphorylation of iRhom2.

ERK1/2 activation is not only induced by oncogenic KRAS but also by several other oncogenes (Liu et al., 2018; Roskoski, 2012; Wee and Wang, 2017), so we asked whether these other ERK1/2-activating oncogenes can similarly drive ADAM17 activity. HRAS^G12V^, BRAF^V600E^ and SRC^Y530F^, all of which activated ERK1/2 (Fig. S2D), also induced elevated release of TGFα from HEK293T cells (Fig. 2D). This result is consistent with the increase in TGFα release seen previously with oncogenic SRC (Maretzky et al., 2011). ERK1/2-activating oncogenes KRAS^G12V^ and BRAF^V600E^ also triggered the release of amphiregulin (Fig. S2E), another ADAM17-dependent ERBB ligand with a well-established role in oncogenesis (Busser et al., 2011; Xu et al., 2016). This contrasted with no increase of phosphorylated (p)ERK levels (Fig. S2D) (Beaver et al., 2013; Lauring et al., 2010) and a very small increase in amphiregulin release (Fig. S2E) caused by the oncogene AKT1^E17K^. These results suggest that the ability of ERK1/2-activating oncogenes to trigger the release of ERBB ligands depends on a common mechanism driven by phosphorylated iRhom2.

### Cancer-associated mutations in iRhom2 potentiate KRAS-induced shedding of ERBB ligands

**Fig. 3.**
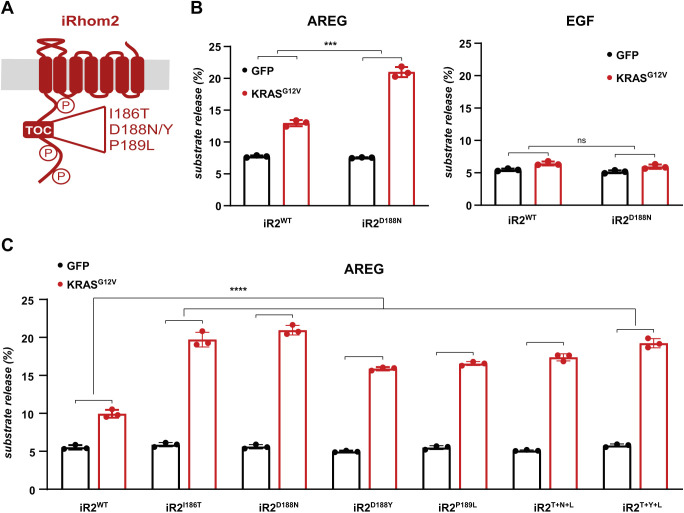
**Cancer-associated mutations in iRhom2 potentiate KRAS-induced shedding of ERBB ligands.** (A) Schematic of the N-terminal domain of iRhom2 with the three main sites of phosphorylation and the conserved region that harbours mutations causing tylosis with oesophageal cancer (TOC). The four analysed TOC mutations are shown in the expansion. (B,C) iRhom1/2 DKO HEK293T cells were reconstituted with iRhom2^WT^ or with an iRhom2 variant harbouring one of the TOC mutations or the three mutations combined: T+Y+L (I186T, D188Y, P189L) or T+N+L (I186T, D188N, P189L). Upon co-transfection with GFP or GFP-tagged KRAS^G12V^, and alkaline phosphatase (AP)-tagged AREG or EGF, overnight collection of medium was performed in biological triplicates. Substrate release is the level of released alkaline phosphatase in the medium divided by the total alkaline phosphatase level. Error bars represent s.d. ***P<0.001; ****P<0.0001; ns, not significant (*P*>0.05) for the induction ratio (KRAS^G12V^/GFP) [unpaired two-tailed Student's *t*-test (B) or one-way ANOVA and Tukey multiple comparison test (C)]. In C, the induction ratio of each of the mutants is significantly greater than wild-type iRhom2 (*P*<0.0001).

Our data demonstrate that iRhom2 phosphorylation participates in oncogenic signalling. The strongest and most direct evidence for the involvement of iRhom2 in human cancer is in the case of a rare inherited syndrome called tylosis with oesophageal cancer (TOC), which is caused by mutations in a small and highly conserved region within the cytoplasmic N-terminal domain of iRhom2 (Fig. 3A) (Blaydon et al., 2012). TOC is characterised by hyperkeratosis, oesophageal cancer, and at least in the case of one of the familial mutations, iRhom2^D188N^, by a susceptibility to other cancers (Saarinen et al., 2012). We therefore investigated whether the tylotic mutations affect oncogenic signalling through ADAM17. Replacing wild-type iRhom2 with tylotic iRhom2^D188N^ caused a strong enhancement of KRAS-induced shedding of the ADAM17 substrate and the ERBB ligand amphiregulin (Fig. 3B). The shedding of EGF, which is triggered by ADAM10 rather than ADAM17 (Sahin et al., 2004), is not affected by iRhom2^D188N^ (Fig. 3B), demonstrating the specificity of the oncogenic iRhom2 mutation for ADAM17. Strikingly, all analysed TOC mutations, including when combined, amplified KRAS-induced amphiregulin release (Fig. 3C) and none affected EGF shedding (Fig. S3A). Furthermore, none of the tylotic mutations altered ADAM17 maturation (Fig. S3B), consistent with our previous conclusion that the cytoplasmic tail of iRhom2 does not participate in the earlier iRhom2 function of promoting ER to Golgi trafficking of ADAM17 (Grieve et al., 2017). We conclude that TOC mutations are sufficient to potentiate KRAS-induced shedding of ADAM17 substrates in HEK293T cells, thereby establishing the direct effect of mutations in the N-terminus of iRhom2 in oncogene-driven signalling.

### iRhoms are required for KRAS-driven tumorigenesis

**Fig. 4.**
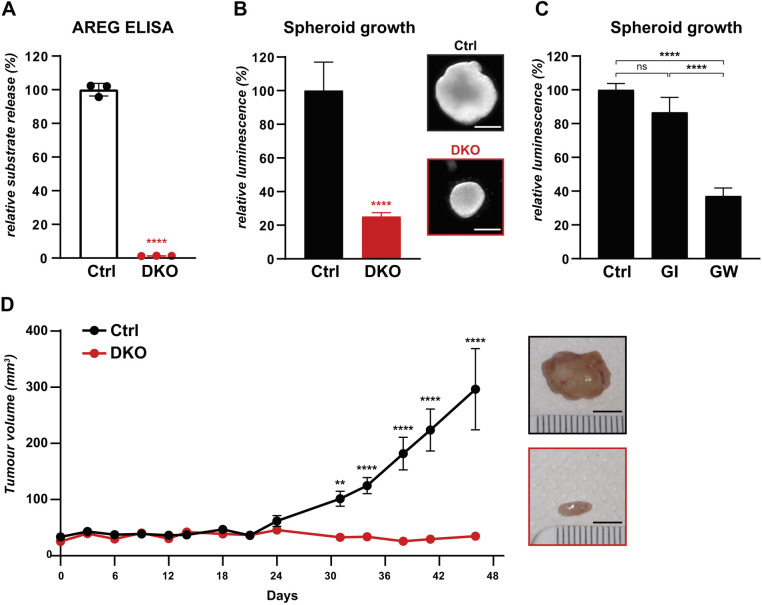
**iRhoms are required for KRAS-driven tumorigenesis.** (A) Release of endogenous amphiregulin (AREG) from control (Ctrl) and iRhom1/2 DKO lung cancer cells A549 was measured after overnight collection in biological triplicates. AREG concentration determined by ELISA was normalised to the total protein concentration in A549 cells, and the average level of A549 Ctrl was defined as the reference (100%). Unless specified otherwise, ELISA experiments are similarly normalised in all experiments. Error bars represent s.d. ***P<0.0001 (unpaired two-tailed Student's *t*-test). (B,C) Spheroid growth of Ctrl and DKO A549 cells in ultra-low attachment plates was performed for 13 days and treated with 2 μM ADAM10 inhibitor (GI) or 2 μM ADAM17 and ADAM10 inhibitor (GW) when indicated. Cell viability quantified using CellTiter Glo was normalised to Ctrl. At least three biological replicates were performed per condition, error bars represent s.d. ****P<0.0001; ns, not significant (*P*>0.05) (unpaired two-tailed Student's *t*-test or one-way ANOVA and Tukey multiple comparison test). Representative spheroids of B are shown as insets. Scale bars: 0.2 mm. (D) Tumour volume of Ctrl and iRhom1/2 DKO A549 xenografts assessed twice weekly, starting 7 days post injection of 10^6^ cells in immunodeficient NSG mice (*n*=6 mice per cell line). Error bars represent s.e.m. **P<0.01; ****P<0.0001 (unpaired two-tailed Student's *t*-test). Insets show representative tumours. Scale bars: 5 mm.

Increased activation of ERBB1/EGFR as well as of the other ERBB receptors have widespread involvement in cancers (Hynes and MacDonald, 2009; Tebbutt et al., 2013; Wang, 2017) including, it has recently been established, in KRAS-induced lung tumorigenesis (Kharbanda et al., 2020; Kruspig et al., 2018; Moll et al., 2018). We therefore addressed the potential role of iRhoms in A549 cells, a widely used human lung adenocarcinoma cell model. These cells were selected because they are homozygous for *KRAS^G12S^*, one of the mutations that we have shown drives TGFα release (Fig. 1C). Using CRISPR/Cas9, we knocked out both *iRhom1* and *iRhom2* in A549 cells to create three A549-DKO clonal cell lines, each of which, as expected, lacked ADAM17 maturation (Fig. S4A). The loss of iRhom activity, and thus of sheddase activity, was confirmed by the impairment shedding of the endogenous ERBB ligand amphiregulin (Fig. 4A; Fig. S4B). Unless otherwise specified, A549-DKO will from now on refer to A549 DKO clone 1. In addition, depletion of iRhoms induced a similar decrease in amphiregulin release in the KRAS mutant NSCLC cells lines NCI-H358 and NCI-H1792 (Fig. S4C), demonstrating the conserved function of iRhoms in promoting growth factor signalling in lung cancer cell lines. In support of this conclusion, A549-DKO cells also showed a decrease in cell proliferation (Fig. S4D).

We next assayed the requirement for iRhoms in the growth of A549 spheroids, three-dimensional (3D) models of solid tumours (Gilazieva et al., 2020; Ham et al., 2016). Supporting the significance of the standard two-dimensional (2D) cell culture result (Fig. S4D), loss of iRhom1 and iRhom2 also significantly inhibited spheroid growth (Fig. 4B; Fig. S4E). Consistent with the implication that iRhom-induced release of ERBB ligands contributes to spheroid growth, inhibition of ADAM17 but not ADAM10 also inhibited growth (Fig. 4C; Fig. S4F).

These results prompted us to ask whether iRhoms also participate in tumorigenesis *in vivo*, using a xenograft model in which A549 cells are injected into immunodeficient mice. This xenograft model allows preclinical evaluation of the role of candidate target genes in tumour formation and maintenance (Gengenbacher et al., 2017). We established xenografts of A549 parental cells and A549-DKO cells, and found that loss of iRhoms had a profound effect, preventing all detectable tumour growth (Fig. 4D). Based on the results in A549 lung cancer cells in 2D cell culture, 3D spheroid growth and tumour xenograft, we conclude that iRhoms are required for oncogenic signalling and lung tumour growth.

### iRhom2 phosphorylation regulates ADAM17-dependent release of ERBB ligand and tumour spheroid growth in lung cancer cells

**Fig. 5.**
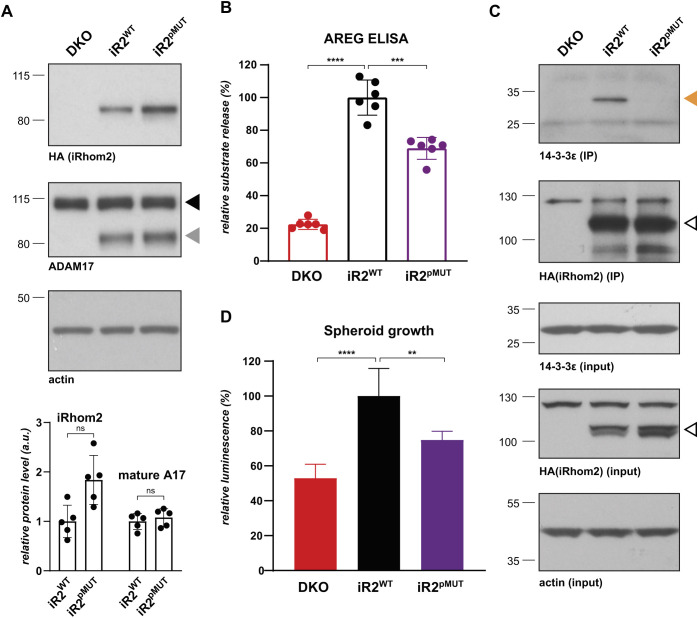
**iRhom2 phosphorylation regulates ADAM17-dependent release of ERBB ligand and tumour spheroid growth in lung cancer cells.** (A) iRhom1/2 DKO A549 cells reconstituted with HA-tagged iRhom2^WT^ or phosphomutant iRhom2 (iRhom2^pMUT^) were immunoblotted for HA, ADAM17 and β-actin. Grey and black arrowheads indicate mature and immature ADAM17, respectively. iRhom2 and mature ADAM17 levels from five biological replicates were quantified relative to the β-actin level using ImageJ. Error bars represent s.d. ns, not significant (*P*>0.05) (unpaired two-tailed Student's *t*-test). (B) Release of endogenous amphiregulin (AREG) from DKO A549 parental cells, or those stably expressing iRhom2^WT^ or iRhom2^pMUT^ was measured in six biological replicates by ELISA after overnight collection and normalised as described previously. Error bars represent s.d. ***P<0.001; ****P<0.0001 for log transformed data (one-way ANOVA and Tukey multiple comparison test). (C) HA immunoprecipitates and lysates from A549 DKO cells stably expressing HA-tagged iRhom2^WT^ or iRhom2^pMUT^, immunoblotted for 14-3-3ε, HA and actin. Orange and open arrowheads indicate 14-3-3ε and HA-tagged iRhom2 constructs, respectively. This experiment was performed in biological triplicates. (D) Spheroid growth of DKO A549 parental cells, or those stably expressing iRhom2^WT^ or iRhom2^pMUT^ was measured after 14 days in five biological replicates by CellTiter Glo and normalised as described previously. Error bars represent s.d. **P<0.01; ****P<0.0001 (one-way ANOVA and Tukey multiple comparison test).

Having established that iRhoms are required in a lung tumorigenesis model, we addressed the molecular mechanism that underlies the pro-tumorigenic function of iRhom2 in A549 cells, using our earlier work in HEK293T cells as a guide. First, we made a phosphomutant version of iRhom2 in which the three main phosphorylation sites (site1-3) as well as additional contributing sites were mutated to alanine residues (iRhom2^pMUT^, Fig. S2A). Although iRhom2^pMUT^ efficiently rescued ADAM17 maturation (Fig. 5A) and thus the basal shedding activity, it significantly inhibited the release of endogenous amphiregulin from A549 cells (Fig. 5B), demonstrating that the phosphorylation-specific function of iRhom2 is required to trigger the full release of growth factors by ADAM17. Second, ERK1/2 kinases drive this mechanism, as evidenced by our observation that the inhibitor U1026 blocked amphiregulin release (Fig. S5). Third, phosphorylation of iRhom2 is required for 14-3-3 binding in A549 cells (Fig. 5C), indicating that the phosphorylated iRhom2/14-3-3/ADAM17 activation pathway controls shedding of the ERBB ligands in these lung cancer cells. Together with our results in HEK293T cells, these results support the conclusion that oncogenic KRAS drives ERBB signalling by inducing iRhom2 phosphorylation. We investigated the biological importance of phosphorylation in iRhom2-dependent shedding of ERBB ligand by performing the 3D spheroid assay, which showed that spheroid growth of reconstituted A549-DKO cells was significantly reduced in iRhom2^pMUT^-expressing cells, compared to what was seen in cells expressing iRhom2^WT^ (Fig. 5D). Overall, our results highlight the pro-tumorigenic role of iRhom2-dependent shedding of ERBB ligands in lung cancer cells.

### Cancer-associated mutations in iRhom2 increase RAS activity and drive a positive feedback loop in lung cancer cells

**Fig. 6.**
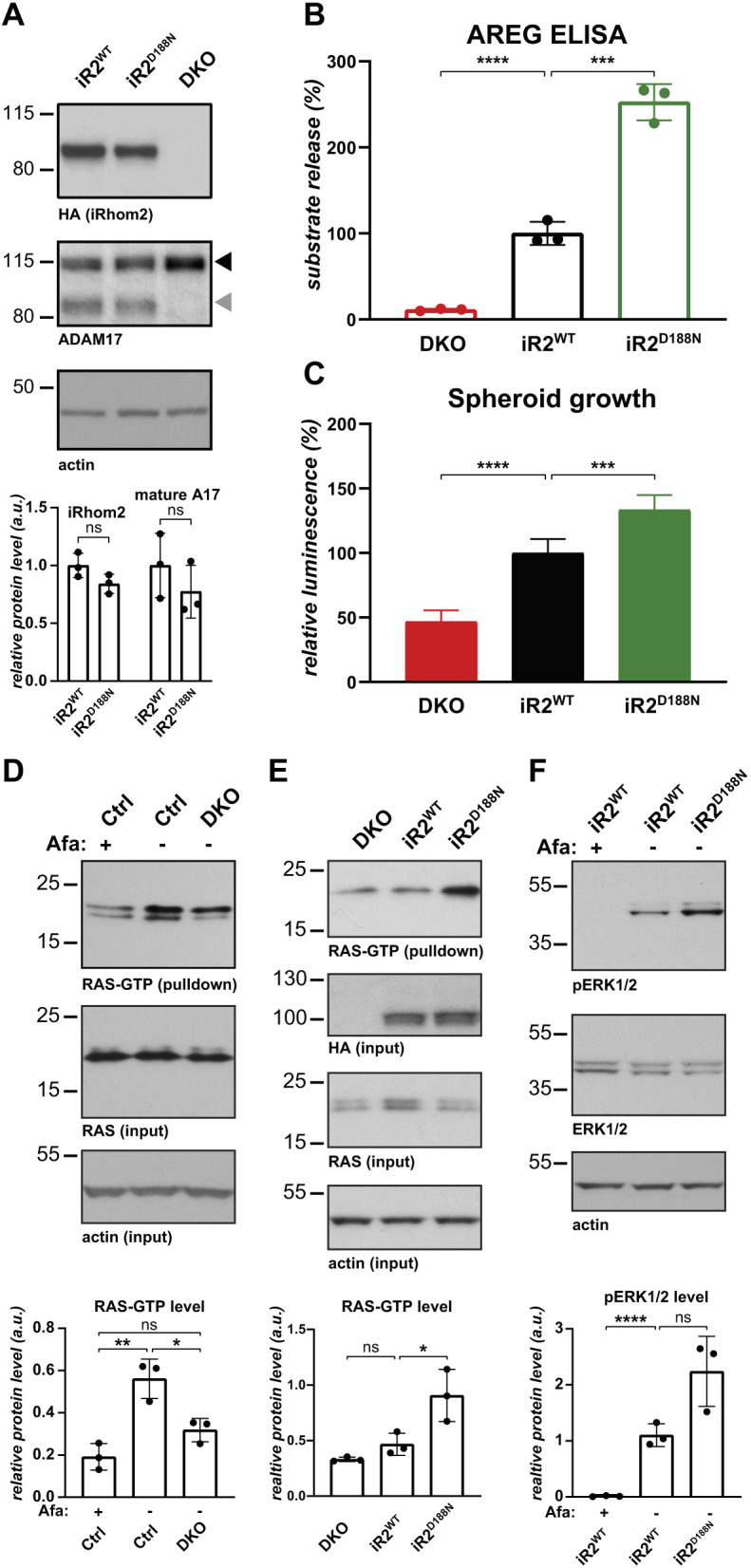
**Cancer-associated mutations in iRhom2 increase RAS activity and drive a positive feedback loop in lung cancer cells.** (A) iRhom1/2 DKO A549 cells reconstituted with HA-tagged TOC iRhom2^D188N^ or iRhom2^WT^ were immunoblotted for HA, ADAM17 and β-actin. Grey and black arrowheads indicate mature and immature ADAM17, respectively. iRhom2 and mature ADAM17 levels from three biological replicates were quantified relative to β-actin level using ImageJ. Error bars represent s.d. ns, not significant (*P*>0.05) (unpaired two-tailed Student's *t*-test). (B) Release of endogenous amphiregulin (AREG) from DKO A549 parental cells, or those stably expressing iRhom2^WT^ or iRhom2^D188N^, was measured in three biological replicates by ELISA after overnight medium collection and normalised as described previously. Error bars represent s.d. ***P<0.001, ****P<0.0001 for log transformed data (one-way ANOVA and Tukey multiple comparison test). (C) Spheroid growth of DKO A549 parental cells, or those stably expressing iRhom2^WT^ or iRhom2^D188N^, was measured after 14 days in five biological replicates by CellTiter Glo and normalised as described previously. Error bars represent s.d. ***P<0.001, ****P<0.0001 (one-way ANOVA and Tukey multiple comparison test). (D,E) Active RAS was assayed in Ctrl, iRhom1/2 DKO parental A549 cells or DKO cells stably expressing HA-tagged iRhom2^WT^ or TOC iRhom2^D188N^ through RAS-GTP pulldown. Cells were treated with 1 μM the pan-ERBB inhibitor afatinib (Afa) for 20 h when indicated, and immunoblotted for RAS, HA or β-actin. The experiments were performed in biological triplicates. Active RAS-GTP level from three biological replicates was quantified relative to β-actin level using ImageJ. Error bars represent s.d. *P<0.05; **P<0.01; ns, not significant (*P*>0.05) on untransformed (D) or log transformed data (E) (one-way ANOVA and Tukey multiple comparison test). (F) Conditioned medium from iRhom1/2 DKO A549 cells stably expressing iRhom2^WT^ or TOC iRhom2^D188N^ was used to stimulate the ERBB1/EGFR reporter cell line A431 treated with 1 μM afatinib when indicated. Following stimulation, A431 cells were immunoblotted for ERK1/2, phosphorylated ERK1/2 (pERK1/2) and β-actin. The experiment was performed in biological triplicates. pERK1/2 level from three biological replicates was quantified relative to β-actin using ImageJ. Error bars represent s.d. ****P<0.0001; ns, not significant (*P*>0.05) for log transformed data (one-way ANOVA and Tukey multiple comparison test).

In HEK293T cells, the cancer causing tylotic iRhom2 mutant D188N enhanced amphiregulin release by oncogenic KRAS mutations (Fig. 3). The same experiment in A549 cells confirmed this result in the lung cancer cell line – compared to iRhom2^WT^, expressing iRhom2^D188N^ in A549-DKO cells caused a more than two-fold increase in the release of endogenous amphiregulin in the presence of oncogenic KRAS (Fig. 6A,B), indicating that tylotic mutation sensitises iRhom2 to oncogenic signalling. Strikingly, iRhom2^D188N^ also further increased spheroid growth compared to iRhom2^WT^ (Fig. 6C), demonstrating that even in transformed A549 cells, the elevated release of ERBB ligand caused by the tylotic iRhom2 mutation is sufficient to further promote spheroid growth of lung cancer cells. Importantly, this shows that a single point mutation in the N-terminus of iRhom2 is sufficient to increase the growth of lung cancer cells in spheroids.

Our observation that iRhoms, and in particular tylotic iRhom2^D188N^, induce ERBB signalling, suggests the existence of a positive feedback loop, in which oncogenic KRAS, signalling through iRhom2, ADAM17 and amphiregulin, promotes ERBB activity and ultimately further KRAS activity. This possibility builds on recent results that show that oncogenic KRAS mutations are not fully constitutive: using an allele-specific inhibitor, it has been shown that the activity of a KRAS mutant is modulated by upstream ERBB signalling (Lito et al., 2016; Patricelli et al., 2016). To test this hypothesis, we assayed the activity of oncogenic KRAS in A549 cells by using the RAS-binding domain of Raf1 to pulldown active RAS^GTP^. Compared to parental cells, RAS^GTP^ levels were reduced by the absence of iRhoms in A549-DKO as they were upon treatment with the pan-ERBB inhibitor afatinib (Fig. 6D), thus suggesting that iRhoms are required to maintain RAS activity by activating ERBB signalling. As tylotic iRhom2^D188N^ triggers a strong increase in RAS^GTP^ (Fig. 6E), it further establishes the central role of iRhoms in controlling RAS activity. To definitively conclude whether this feedback loop acts through iRhom2-dependent shedding in the extracellular medium, we assessed the effect of conditioned medium from A549 cells on the ERBB1/EGFR reporter cell line A431. Conditioned medium from tylotic iRhom2^D188N^ caused elevated activated ERK1/2 compared to iRhom2^WT^ (Fig. 6F). We confirmed that iRhom2-driven activation of the Raf/MEK/ERK pathway depends on ERBB signalling by using afatinib (Fig. 6F). Together, these results support that iRhom-dependent shedding of ERBB ligands in the extracellular medium drives a positive feedback loop to maintain the activity of oncogenic KRAS in lung cancer cells.

## DISCUSSION

**Fig. 7.**
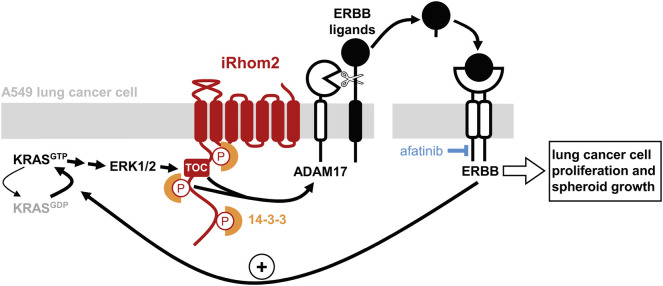
**iRhom activity drives an ERBB-dependent feedback loop on oncogenic KRAS.** Activation of ERK1/2 by oncogenic KRAS^GTP^ triggers the phosphorylation of iRhom2 and subsequent recruitment of the phospho-binding 14-3-3 proteins. Together with mutations responsible for tylosis with oesophageal cancer (TOC), this induces the ADAM17-dependent release of ERBB ligands into the extracellular medium. Upon binding their ligands, ERBBs maintain KRAS in the active GTP-bound state, thus enabling a positive feedback loop for KRAS oncogenesis. This feedback loop can be inhibited by blocking ERBB signalling with afatinib.

We have discovered that, by regulating ADAM17-dependent release of ERBB ligands, iRhoms are required for KRAS-driven tumorigenesis. Oncogenic mutants of KRAS induce ERK1/2-dependent phosphorylation of the cytoplasmic domain of iRhom2, triggering the recruitment of the phospho-binding 14-3-3 proteins, which in turn activate ADAM17 to shed ERBB ligands from the plasma membrane (Fig. 7). The relevance of this mechanism to human disease is demonstrated by our discovery that mutations in the cytoplasmic domain of iRhom2, known to be causative of the human cancer syndrome TOC, are sufficient to amplify this signalling pathway. The significance of iRhom2 to cancer pathogenesis is further reinforced by the result that loss of iRhom activity from A549 lung cancer cells completely blocks their ability to form tumours in a xenograft model.

### iRhom2 is at the centre of a positive feedback loop to sustain oncogenic KRAS activity

As well as identifying iRhom2 as an essential player in KRAS-induced tumorigenesis, these results reveal the existence of a previously unidentified positive feedback loop that maintains RAS activity in lung cancer cells. In agreement with biochemical evidence proving that, contrary to prior belief, activated KRAS mutations are ‘hyperexcitable’ rather than constitutively locked in an active state (Lito et al., 2016; Patricelli et al., 2016), two recent studies have shown that oncogenic KRAS relies on upstream ERBB signalling to remain active, and thus to drive lung tumorigenesis (Kruspig et al., 2018; Moll et al., 2018). Our data establish that the cytoplasmic domain of iRhom2 is crucial in this mechanism; by being both downstream of oncogenic KRAS, and sufficient to increase ERBB-dependent RAS activation, the cytoplasmic domain of iRhom2 represents a central component of this newly uncovered positive feedback loop (Fig. 7). The existence of this feedback mechanism presupposes a sufficient pool of immature, plasma membrane-bound ERBB ligands that can be released in response to elevated iRhom2– ADAM17 activity to reinforce oncogenic KRAS activity. This requirement is supported by the recent observation that the expression of amphiregulin (and other ERBB ligands) is indeed elevated in KRAS-induced lung tumours (Kruspig et al., 2018). Overall, our results strengthen a now compelling body of evidence that overturns the earlier belief that oncogenic KRAS mutations are fully constitutive – instead, it is clear that KRAS-driven tumours are driven by signalling input to the activated KRAS oncoprotein. This opens a new potential strategy for therapeutic intervention.

### iRhom2 induces activation of ADAM17 through a conserved mechanism in immune and cancer cells

Our results demonstrate that oncogenic and inflammatory signalling pathways share a conserved mechanism for the activation of the iRhom2–ADAM17 complex (this study and Cavadas et al., 2017; Grieve et al., 2017). One molecular aspect of the activation of ADAM17 by iRhom2 that we previously reported was that phosphorylation and 14-3-3 binding to iRhom2 causes some kind of conformational change in the complex between the two proteins, detected by weaker binding between them (Grieve et al., 2017). This partial uncoupling also occurred during KRAS-induced shedding (Fig. 2C), thus further demonstrating the conserved activation of the iRhom2–ADAM17 complex. Finally, the growing number of functional signalling complexes in which iRhom2 participates – iRhom2 and ADAM17 (Adrain et al., 2012; Cavadas et al., 2017; Grieve et al., 2017; McIlwain et al., 2012), iRhom2 and KRAS (Fig. 2C; Go et al., 2021), and iRhom2 and the previously described binding partner FRMD8 (Künzel et al., 2018; Oikonomidi et al., 2018) – strengthen the incentives to adopt mechanistic and structural approaches to understanding how iRhom2 controls ADAM17 signalling. For example, it would be interesting to investigate whether, in addition to 14-3-3, other iRhom2 interactors participate in KRAS-driven lung tumour formation.

### Phosphorylation of the iRhom2–ADAM17 complex as an essential driver of KRAS-induced lung tumorigenesis

In work that complements these results, Saad et al. reported the requirement of ADAM17 in KRAS-induced lung tumorigenesis. They demonstrated that oncogenic KRAS induces the phosphorylation of ADAM17, as well as the release of its substrate IL-6R and an increase of ERK1/2 activation (Saad et al., 2019). Their work emphasises the need for better understanding of the potential role of the phosphorylation of ADAM17 on its catalytic activity (Düsterhöft et al., 2019; Zunke and Rose-John, 2017). Together with our work that demonstrates a clear molecular mechanism for the role of iRhom2 phosphorylation in the release of ERBB ligands, this establishes the wider significance of regulated shedding by ADAM17 as a mediator of oncogenic KRAS signalling. It will be interesting to explore the differences and possible crosstalk between the systems that lead, on one hand to shedding of soluble IL-6R triggered by phosphorylated ADAM17, and on the other hand, to ERBB ligand shedding induced by phosphorylated iRhom2.

### The iRhom2–ADAM17 complex in other cancer types

Oncogenic KRAS is a driver of multiple cancers in addition to lung adenocarcinoma, so our work raises the question of whether iRhom2 also has a role in these other cancers. Pancreatic adenocarcinoma, the seventh leading cause of cancer-related deaths worldwide, is considered the most KRAS-addicted cancer (Bray et al., 2018; Rawla et al., 2019b; Waters and Der, 2018). Strikingly, ADAM17 and ERBB1/EGFR are both required to maintain high RAS activity in a *Kras^G12D^* mouse model of pancreatic ductal carcinoma (Ardito et al., 2012; Navas et al., 2012). In the light of the results we report here, it will be interesting to investigate whether iRhom2 plays a similar role in supporting a positive feedback loop in this particularly aggressive oncogenic context. In support of this possibility, the cytoplasmic domain of iRhom2 has been found to be phosphorylated in the presence of KRAS^G12D^ in pancreatic cancer cells (Tape et al., 2016). Another case where there is now a strong incentive to explore the possible involvement of iRhom2 is colorectal cancer, the second leading cause of cancer-related deaths worldwide (Bray et al., 2018; Rawla et al., 2019a), which can also be driven by oncogenic KRAS mutations (Cicenas et al., 2017). Using patient-derived organoids and xenografts, it has recently been demonstrated that ERBB signalling promotes tumorigenesis by maintaining ERK activity in colorectal tumours (Ponsioen et al., 2021). Although ADAM17 has been shown to be required for colorectal tumour growth (Schmidt et al., 2018), the possible contribution of iRhom2 phosphorylation in colorectal tumorigenesis is currently unexplored. Finally, further work will be required to characterise the molecular mechanism by which TOC mutations trigger activation of ADAM17. For example, we speculate that TOC mutations might finetune the active iRhom2–ADAM17 complex, or that they affect the recruitment of the cytosolic effectors of iRhom2.

In summary, we have shown that by driving ADAM17-dependent ERBB signalling, iRhoms are essential components in KRAS-driven tumorigenesis. On a mechanistic level, we report the existence of a KRAS/iRhom2/ERBB positive feedback loop that maintains oncogenic KRAS activity and might explain the potency of KRAS-induced cancers. Finally, by establishing the role of iRhom2 in oncogenic activation of ADAM17, our results provide new routes to explore future therapeutic opportunities.

## MATERIALS AND METHODS

### Molecular cloning

iRhom2, KRAS4A, KRAS4B SRC, BRAF and AKT1 constructs were amplified by PCR from *iRhom2* cDNA (Künzel et al., 2018), *KRAS4A* cDNA (Grieve and Rabouille, 2014), *KRAS4B* cDNA (a kind gift from Julian Downward, Francis Crick Institute, London), *SRC* cDNA (antibodies-online), *BRAF* cDNA (antibodies-online) and *AKT1* cDNA (antibodies-online). They were mutated using the QuikChange Multi Site-Directed Mutagenesis Kit (Agilent Technologies, 200515) and subcloned using In-Fusion HD Cloning Kit (Takara Bio, 639649) according to the manufacturer's instructions. For all constructs, single colonies were picked and extracted DNA was verified by Sanger sequencing (Source Bioscience, Oxford, UK). The list of the plasmids used in the study is available in Table S1.

### Cell culture and transfections

Human embryonic kidney (HEK) 293T cells and human non-small-cell lung cancer (NSCLC) A549 cells were cultured in DMEM (Sigma-Aldrich) supplemented with 10% fetal bovine serum (FBS) (Sigma-Aldrich) and 2 mM L-glutamine (Gibco) at 37°C with 5% CO_2_. Human carcinoma A431 cells were cultured in EMEM (Lonza) supplemented with 10% FBS and 2 mM L-glutamine (Gibco). FuGENE HD (Promega) was used for transient DNA transfection in HEK293T cells, with a ratio of 1 μg DNA and 4 μl transfection reagent diluted in OptiMEM (Gibco). Lipofectamine 2000 (Thermo Fisher Scientific) was used for transient DNA transfection of A549 cells, with a ratio of 0.3 μg DNA and 1 μl transfection reagent. The knockdown experiments in NSCLC cell lines were performed with 60 nM siRNA using Lipofectamine RNAiMAX (Thermo Fisher Scientific) following the manufacturer's instructions. The list of the siRNAs used in the study is available in Table S2. The HEK DKO cells stably expressing pLVX-TetOne-zeo constructs were stimulated with 100 ng/ml doxycycline (MP Biomedicals, 195044).

### CRISPR/Cas9 genome editing in A549 and HEK293T cells

CRISPR/Cas9-mediated single knockout of human *RHBDF1* (encoding *iRhom1*) or *RHBDF2* (encoding iRhom2) in HEK293T was performed as described previously (Künzel et al., 2018), and the list of the primers used in the study is available in Table S3. In brief, the plasmids co-expressing Cas9 nickase (Cas9n) and the gRNA targeting *RHBDF1* or *RHBDF2* were transfected into HEK293T cells. Upon puromycin selection and isolation of single colonies, the loss of *RHBDF1* or *RHBDF2* was analysed by PCR.

For CRISPR/Cas9-mediated double knockout of human *RHBDF1* and *RHBDF2* in A549 cells, 4 μg of plasmids co-expressing Cas9n and the gRNA were transfected using the Neon Transfection System (Invitrogen) according to the manufacturer's instructions. The following electroporation settings were used: 1230 volts, 30 s pulse width, 2 pulses number and 8×10^6^ cells/ml. Antibiotic selection was performed using 0.5 μg/ml puromycin for 48 h, before selecting single colonies to establish clonal cell lines, and analysing loss of *RHBDF1* and *RHBDF2* by PCR.

### Lentiviral transduction of cell lines

A549 or HEK293T DKO cells stably expressing iRhom2 constructs were generated by lentiviral transduction using the pLVX-TetOne or pHRSIN constructs as previously described (Adrain et al., 2012). Cells were selected by adding 50 μg/ml zeocin or 10 μg/ml Blasticidin S HCl. The list of the cell lines used in the study is available in Table S4.

### Co-immunoprecipitation

Cells were washed three times with ice-cold PBS before lysis in Triton X-100 lysis buffer (1% Triton X-100, 150 mM NaCl, 50 mM Tris-HCl pH 7.5) supplemented with EDTA-free complete protease inhibitor mix (Roche, 11873580001), 10 mM 1,10-phenanthroline (Sigma-Aldrich, 131377-5G) and PhosSTOP (Roche, 04906837001). Pre-washed anti-HA magnetic beads (Thermo Fisher Scientific, 88837) were added to the lysates cleared from cell debris by centrifugation at 21,000 ***g*** at 4°C for 15 min and incubated for at least 2 h on a rotor at 4°C. Beads were washed five times with Triton X-100 lysis buffer and eluted with a 10-min incubation at 65°C in 2× SDS sample buffer (0.25 M Tris-HCl pH 6.8, 10% SDS, 50% glycerol, 0.02% bromophenol blue) supplemented with 200 mM DTT.

### Concanavalin A enrichment

Cell lysates were incubated with 30 μl concanavalin A–Sepharose (Sigma-Aldrich, C9017-25ML) at 4°C for 2 h on a rotor. Beads were pelleted at 1500 ***g*** for 2 min at 4°C and washed five times with Triton X-100 lysis buffer. Glycoroteins were eluted with 2× LDS buffer (Invitrogen) supplemented with 25% sucrose and 50 mM DTT for 10 min at 65°C.

### RAS-GTP pulldown

To detect active RAS in A549 cells, RAS-GTP pulldown was performed according to the manufacturer's instructions using the Active Ras Detection Kit (Cell Signaling Technology, #8821). In brief, one confluent 10 cm dish of cells was rinsed with ice-cold PBS and lysed in 0.5 ml ice-cold lysis buffer supplemented with 1 mM PMSF. Cell lysates were cleared by centrifugation (16,000 ***g*** for 15 min at 4°C) and protein concentration was determined by Bradford assay. Cleared lysates were added to the pre-washed spin cup which contains 100 μl of the 50% resin slurry and 80 μg of GST-Raf1-RBD and incubated at 4°C for 1 h on a rotor. The resin was washed three times with Wash Buffer and the proteins bound to the resin were eluted with 50 μl of the sample buffer supplemented with 200 mM DTT. Samples were denatured at 95°C for 5 min and were subjected to western blot analysis.

### SDS-PAGE and western blotting

Cells were washed with ice-cold PBS before lysis in Triton X-100 lysis buffer supplemented with EDTA-free complete protease inhibitor mix (Roche, 11873580001), 10 mM 1,10-phenanthroline (Sigma-Aldrich, 131377-5G) and, when blotting for phosphoproteins, with PhosSTOP (Roche, 04906837001). Cell lysates were denatured at 65°C for 10 min in sample buffer supplemented with 100 mM DTT. Samples were run in 4–12% Bis-Tris NuPAGE gradient gels (Invitrogen) and MOPS running buffer (50 mM MOPS, 50 mM Tris, 0.1% SDS, 1 mM EDTA, pH 7.7), or in Novex 8-16% Tris-Glycine Mini Gels with WedgeWell format (Thermo Fisher Scientific) and Tris-glycine running buffer (25 mM Tris base, 192 mM glycine, 0.1% SDS, pH 8.3). Proteins were then transferred to a methanol-activated polyvinylidene difluoride (PVDF) membrane (Millipore) in Bis-Tris or Tris-Glycine transfer buffer. 5% milk in PBST (0.1% Tween 20) or TBST (0.05% Tween 20) was used for blocking and antibody incubation, and PBST or TBST was used for washing. The membranes were incubated with secondary antibodies at the room temperature for 1 h. Blots were quantified using ImageJ. The list of the antibodies used in this study is available in Table S5, and images of uncropped western blots are shown in Fig. S6.

### AP-shedding assay

HEK293T cell lines were seeded in poly-L-lysine (PLL, Sigma-Aldrich) coated 24-well plates in triplicate 24 hours before transfection. 50 ng alkaline phosphatase (AP)-conjugated substrates were transfected with FuGENE HD (Promega, E2312). In KRAS-related experiments, 100 ng control plasmids or KRAS plasmids were transfected together with AP substrates. At 24 h after transfection, cells were washed twice with PBS and incubated for 18 h in 300 μl Phenol Red-free OptiMEM (Gibco, 11058-021) supplemented with 1 μM GW280264X (GW; Generon, AOB3632-5) or GI254023X (GI; Sigma, SML0789-5MG) when indicated. For the kinase inhibition assay, 300 μl Phenol Red-free OptiMEM were supplemented with 10 μM U0126 (Abcam, ab120241-5mg). After 3 h incubation, supernatants were collected and cells were lysed in 300 μl Triton X-100 lysis buffer supplemented with EDTA-free protease inhibitor mix (Roche). 100 μl supernatant and 100 μl diluted cell lysates were independently incubated with 100 μl AP substrate p-nitrophenyl phosphate (PNPP; Thermo Fisher Scientific, 37620) at room temperature and the absorbance was measured at 405 nm by a plate reader (SpectraMax M3, Molecular Devices). The percentage of substrate release was calculated by dividing the signal from the supernatant by the total signal (supernatant and cell lysate).

### Spheroid assay

Tumour spheroids were generated as previously described in Molina-Arcas et al. (2019). In brief, 2500 cells were resuspended in culture medium supplemented with 2.5% growth-factor reduced Matrigel (Scientific Laboratory Supplies, #356231) and placed in a 96-well round-bottom ultra-low attachment plate (Corning, #7007). Formation of the spheroids was initiated by centrifugation at 300 ***g*** for 4 min. After 13 days, tumour spheroids were imaged using a stereoscopic microscope (Leica DFC310 FX), and cell viability was measured using the CellTiter-Glo Cell viability assay (Promega, #G9681) according to the manufacturer's instructions.

### Cell proliferation assay

To assay cell proliferation in a 2D adherent format, 1000 cells were seeded in standard 96-well tissue culture plate. After 5 days, cell viability was measured using the CellTiter-Glo Cell viability assay (Promega, #G9681) according to the manufacturer's instructions, as previously described in Patricelli et al. (2016).

### A431 ERBB1/EGFR activation assay

1.5×10^6^ A549 or 3×10^6^ A431 cells were seeded in a 10 cm tissue culture dish. After 3 days, A431 cells were washed once with PBS, and serum-starved in 10 ml of OptiMEM supplemented with 1 μM afatinib (Stratech, A8247-APE) when indicated, and with 2 μM GW and to prevent growth factor release from A431 cells. The following day, the medium of A431 cells was renewed with OptiMEM supplemented with the same inhibitors, while A549 cells were washed once with PBS before adding 5 ml OptiMEM constituting the conditioned medium. After 4 h of collection, A431 cells were incubated with the conditioned medium for 3 min before being placed on ice and lysed as described in the SDS-PAGE and western blotting section.

### ELISA

80,000 A549 cells were seeded in triplicate per well of a 24-well plate. To study the loss of shedding in A549-DKO and A549-DKO-iRhom2^pMUT^ cells, the medium was replaced the following day with 350 μl of full medium (DMEM supplemented with FBS and L-glutamine) and collected after 18 h of incubation. To determine the increased shedding in A549-DKO-iRhom2^D188N^, a 4-h collection was performed 48 h after seeding the cells. Similarly, a 4-h collection in 350 μl of full medium supplemented with 10 μM U0126 was performed to determine the contribution of ERK1/2. In all cases, the concentration of amphiregulin in the supernatant was determined using the Human Amphiregulin Quantikine ELISA Kit (R&D Systems, DAR00) according to the manufacturer's instructions. In parallel, the cells were lysed in Triton X-100 lysis buffer and the total protein concentration was measured using the BCA Assay (Life Technologies). The substrate release was determined by normalising the amphiregulin concentration by the total protein concentration.

### Xenograft model

10^6^ Ctrl and iRhom1/2 double-knockout (DKO) A549 cells were resuspended in Matrigel:PBS (50:50 v/v) before being subcutaneously injected in one flank of 12 (*n*=6 mice per cell line) 6-week-old female immunodeficient NOD.*Cg-Prkdc^scid^Il2rg^tm1Wjl^*/SzJ (NSG) mice (Charles River UK Ltd, Margate, Kent, UK). Xenograft growth was monitored with a calliper twice weekly; tumour volume was determined using the following formula: (length×width^2^)/2. At the end of the experiment, tumours were collected and photographed. Animal experiments were performed under the Home Office Project Licence PPL30/3395 (licence holder, A.J.R.).

### Statistical analysis

Statistics were performed in R (version 4.1.2). The required assumptions for one-way ANOVA were tested for each statistical test. Bartlett's test was used to evaluate the variance homoscedasticity and Shapiro–Wilk test was used to verify residual normality. A log_10_ transformation was applied when heteroscedasticity or non-normal distribution of residuals was detected. Pairwise comparison was performed using a post-hoc Tukey's test.

## Supplementary Material

Supplementary information

Reviewer comments
