# Supplementary material for: iRhom2 regulates ERBB signalling to promote KRAS-driven tumour growth of lung cancer cells
Source: J Cell Sci. 2022 Sep 8;135(17):jcs259949. doi: 10.1242/jcs.259949 (PMC9482348; doi:10.1242/jcs.259949)
Supplement: Supplementary information [file joces-135-259949-s1.pdf]

Fig. S1

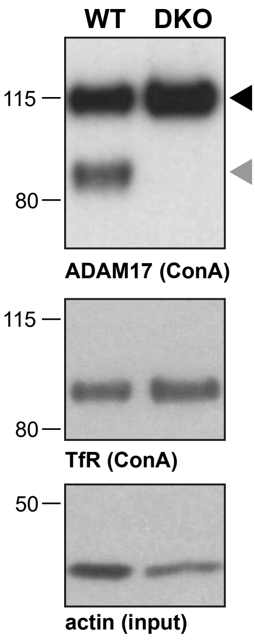

Fig. S1. (Related to Fig. 1) Loss of iRhom activity in HEK293T DKO

Concanavalin A (ConA) enrichment of lysates prepared from iRhom1/2 DKO or WT HEK293T and immunoblotted for ADAM17, transferrin receptor (TfR) or beta-actin. The lack of mature ADAM17 (grey arrowhead), compared to immature proADAM17 (black arrowhead), in the absence of iRhoms demonstrates the loss of all iRhom activity. This experiment was repeated three times.

Journal of Cell Science • Supplementary information

Fig. S2

A

																Reference
mouse iRhom2	S58	S60	S83	S85	S87	S99	S147	Y199	S293	S295	S298	S357	S359	S360	T361	(Grieve et al., 2017)
human iRhom2	S88	S90	S113	S115	S117	S129	S177	Y229	S323	S325	S328	S385	S387	S388	T389	(this paper)
	site1		site2									site3				

Sites mutated in iRhom2^{pMUT}

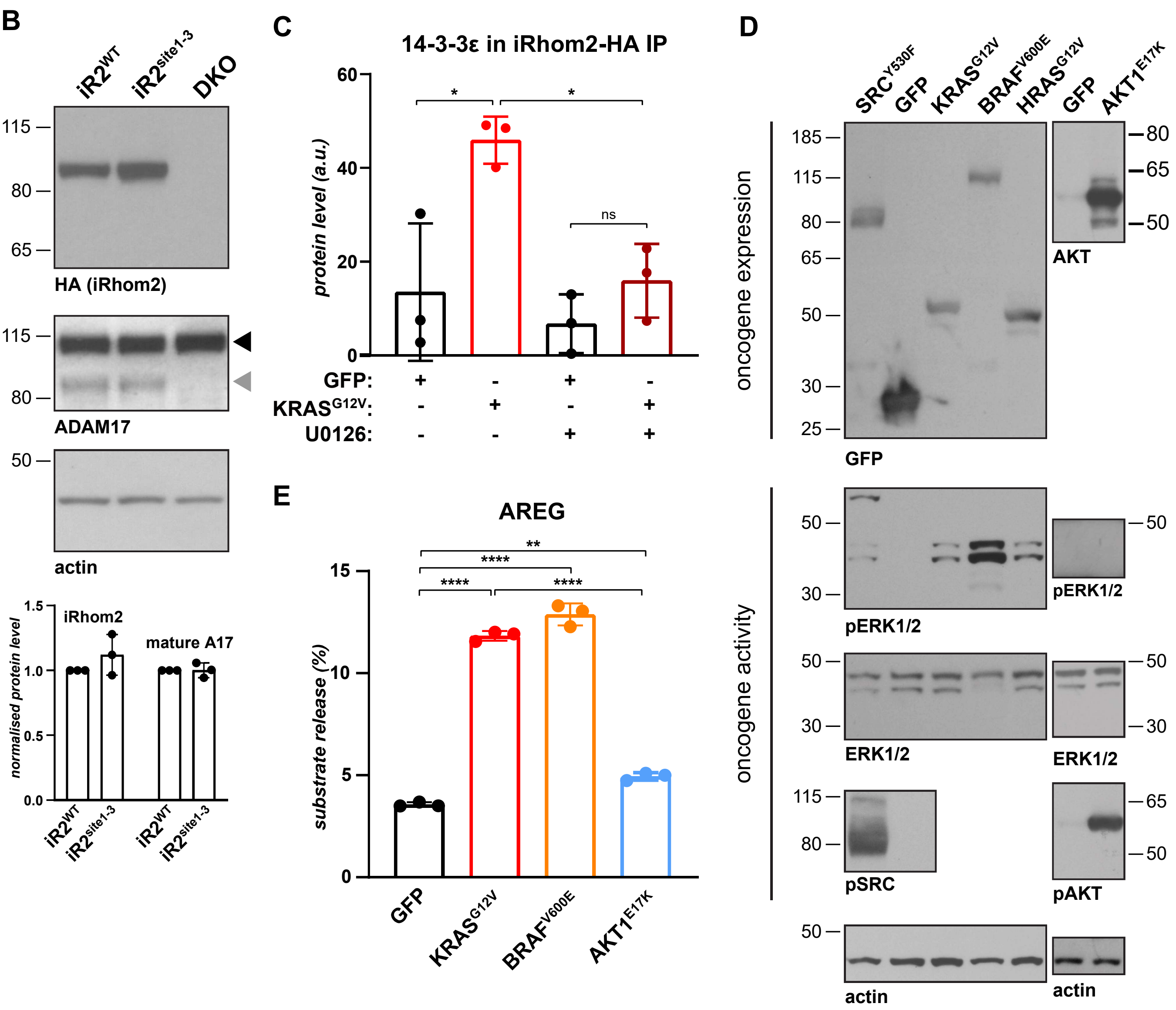

Journal of Cell Science • Supplementary information

Fig. S2. (Related to Fig. 2) KRAS-induced shedding depends on the phosphorylation of the cytoplasmic domain of iRhom2 by the Raf/MEK/ERK pathway

A. Table of phosphorylation sites mutated in $iRhom2^{site1-3}$ and $iRhom2^{pMUT}$. The three critical sites for 14-3-3 binding as well as additional residues contributing to ADAM17 activity were characterised in (Grieve et al., 2017) and mutated to alanine in the human $iRhom2^{pMUT}$ construct.
B. Lysates from iRhom1/2 DKO HEK293T cells reconstituted with HA-tagged $iRhom2^{WT}$ or $iRhom2^{site1-3}$ were immunoblotted for HA, ADAM17 and beta-actin. Grey and black arrowheads indicate mature and immature ADAM17 respectively. iRhom2 and mature ADAM17 levels from at least three biological replicates were quantified relative to beta-actin level using ImageJ.
C. Quantification of 14-3-3ε level in iRhom2-HA IP described in Fig. 2C from three biological replicated. Error bars represent standard deviations and statistical tests were performed using one-way ANOVA and Tukey multiple comparison test. ns = p value>0.05, * = p value <0.05.
D. HEK293T cells transfected with GFP or GFP-tagged SRCY530F, $KRAS^{G12V}$, $BRAF^{V600E}$, $HRAS^{G12V}$, untagged $AKT1^{E17K}$ were immunoblotted for oncogene expression (GFP and AKT1), induction of phosphorylated ERK1/2 (pERK1/2) or for beta-actin. The level of phosphorylated SRC and AKT were probed as a control of their constitutive activity. The experiment was performed in biological triplicates.
E. HEK293T cells were transiently co-transfected with AP-tagged AREG and GFP or GFP-tagged $KRAS^{G12V}$, $BRAF^{V600E}$, untagged $AKT1^{E17K}$. Overnight medium collection was performed in three biological replicates. Substrate release is the level of released alkaline phosphatase in the medium divided by the total alkaline phosphatase level. Error bars represent standard deviations and statistical tests were performed using one-way ANOVA and Tukey multiple comparison test. ** = p value<0.01, **** = p

Journal of Cell Science • Supplementary information

Fig. S3

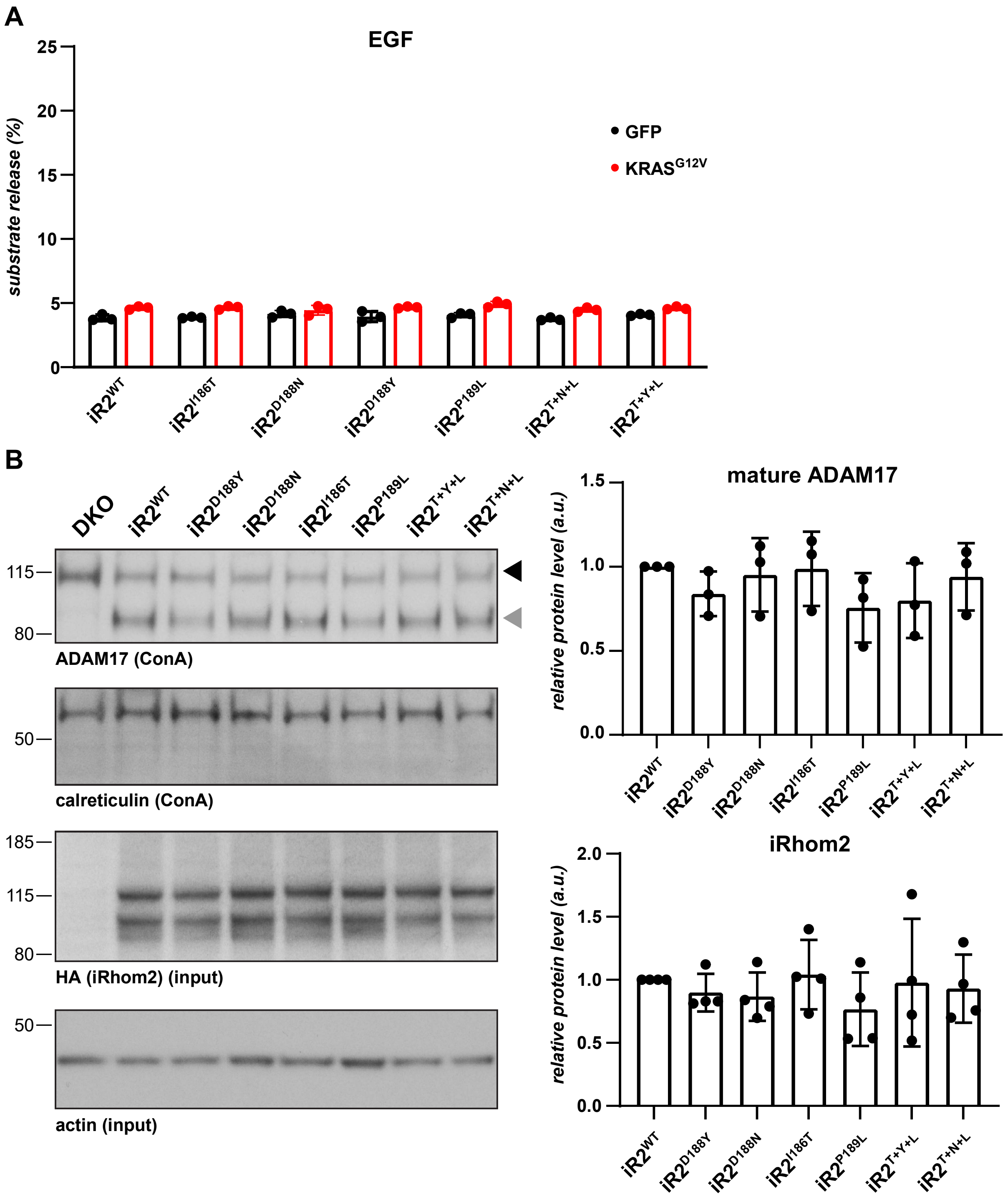

Journal of Cell Science • Supplementary information

Fig. S3. (Related to Fig. 3) Cancer-associated mutations in iRhom2 potentiate KRAS-induced shedding of ERBB ligands

A. iRhom1/2 DKO HEK293T cells reconstituted with iRhom2^{WT} or with an iRhom2 variant harbouring one of the TOC mutations or the three mutations combined: T+Y+L (I186T, D188Y, P189L) or T+N+L (I186T, D188N, P189L) were co-transfected with GFP or GFP-tagged KRAS^{G12V}, and alkaline-phosphatase AP-EGF. Overnight collection of medium was performed in biological triplicates.
B. Concanavalin A (ConA) enrichment of lysates from iRhom1/2 DKO HEK293T cells reconstituted with HA-tagged iRhom2^{WT} or iRhom2 variant harbouring one or a combination of the TOC mutations, followed by immunoblotting for ADAM17 and calreticulin. Black and grey arrowheads indicate immature and mature ADAM17 respectively. Stable expression of HA-tagged iRhom2 variants was detected by HA and beta-actin antibodies. Mature ADAM17 and iRhom2 levels from three biological replicates were quantified using ImageJ relative to total ADAM17 (immature and mature) and beta-actin respectively.

Fig. S4

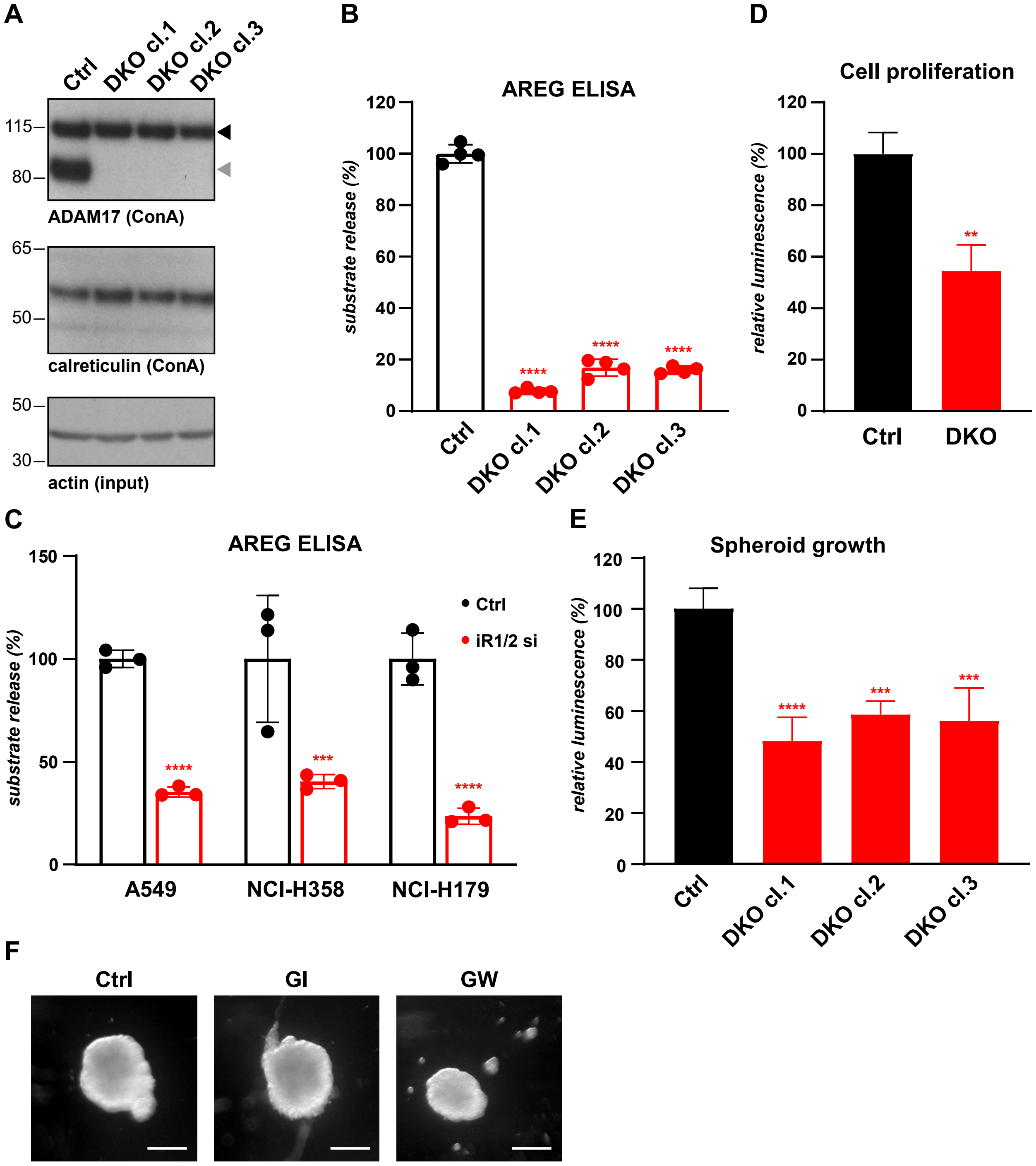

Journal of Cell Science • Supplementary information

Fig. S4. (Related to Fig. 4) iRhoms are required for KRAS-driven tumorigenesis

A. Concanavalin A (ConA) enrichment of lysates from Ctrl and iRhom1/2 DKO A549 clonal (cl.) cell lines, immunoblotted for ADAM17, calreticulin and beta actin. The absence of mature ADAM17 (grey arrowhead) compared to immature proADAM17 (black arrowhead) demonstrates the lack of iRhom activity. The experiment was repeated three times.
B. Release of endogenous AREG from Ctrl or iRhom1/2 DKO clonal A549 cell lines was measured after four hours collection in four biological replicates by ELISA and normalised as described previously. Error bars represent standard deviations and statistical tests were performed using one-way ANOVA and Tukey multiple comparison test. **** = p value<0.0001.
C. Release of endogenous AREG from A549, NCI-H358 and NCI-H1792 KRAS-mutant lung cancer cells lines treated with siRNA negative control (Ctrl) or iRhom1 and iRhom2 stealth siRNA (iR1/2 si) was measured in three biological replicates by ELISA after overnight collection and normalised as described previously. Error bars represent standard deviations and statistical tests were performed using one-way ANOVA and Tukey multiple comparison test. *** = p value<0.001, **** = p value<0.0001.
D. Cell proliferation of Ctrl and iRhom1/2 DKO A549 cells was measured five days after seeding the cells using CellTiter Glo. The luminescence level was normalised to the level of A549 Ctrl (100%). Three biological replicates were performed per cell line, the error bars represent standard deviations and the statistical tests were performed using two-tailed student t-test. ** = p value <0.01.
E. Spheroid growth of Ctrl and DKO A549 clonal cell lines in ultra-low attachment plates was performed for 14 days in four biological replicates. Cell viability quantified using CellTiter Glo was normalised to Ctrl (100%). Error bars represent standard deviations and statistical tests were performed using one-way ANOVA and Tukey multiple comparison test. *** = p value<0.001, **** = p value<0.0001.
F. Representative images of A549 spheroids performed in triplicate (Fig. 4C) are shown after 13 days treatment with 2 µM GI or 2 µM GW when indicated. scale bar = 0.2 mm.

Journal of Cell Science • Supplementary information

Fig. S5

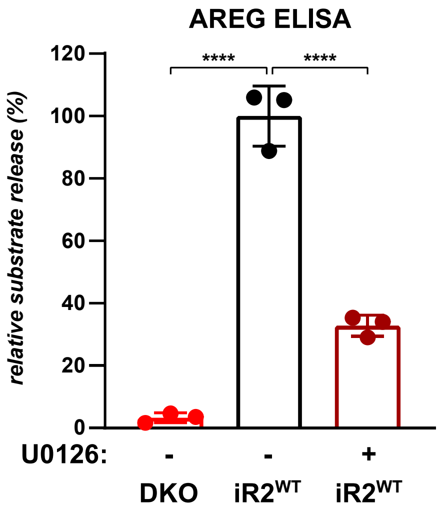

Fig. S5. (Related to Fig. 5) Amphireguling shedding depends on ERK1/2 activity in A549 cells

Release of endogenous AREG from DKO A549 parental cells or those stably expressing iRhom2^{WT}, measured by ELISA after four hours of treatment with DMSO or 10 µM U0126. Substrate release was normalised as previously described, error bars represent standard deviations and statistical tests were performed using one-way ANOVA and Tukey multiple comparison test. **** = p value<0.0001.

Journal of Cell Science • Supplementary information

Fig. S6 (Blot transparency)

Fig. 2C

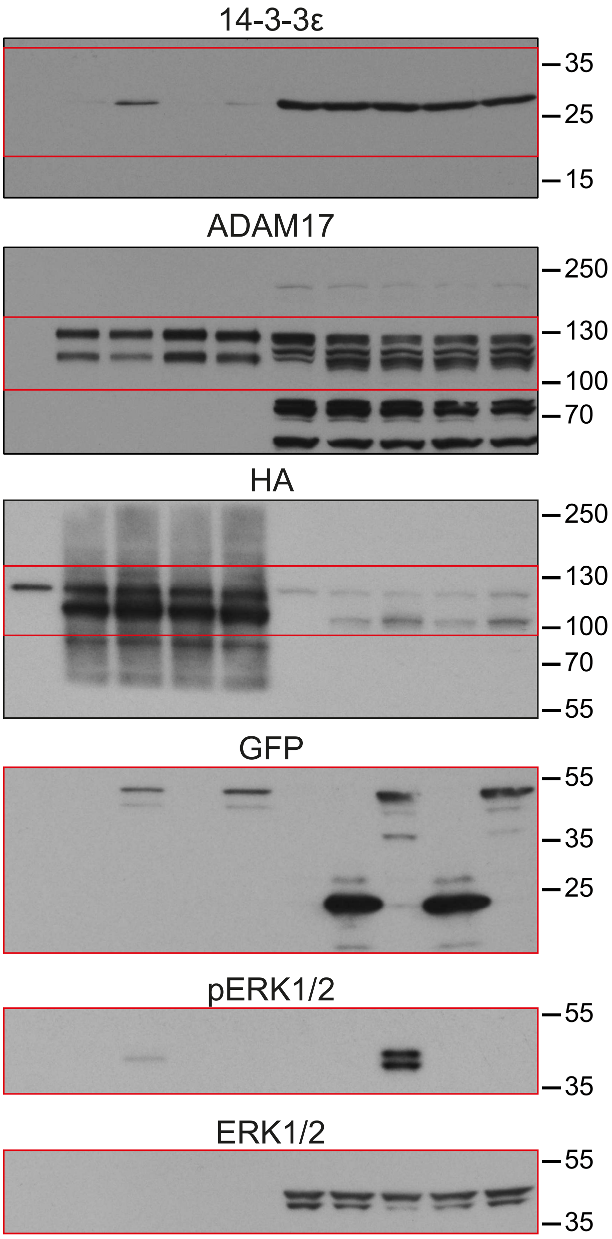

Fig. 5A

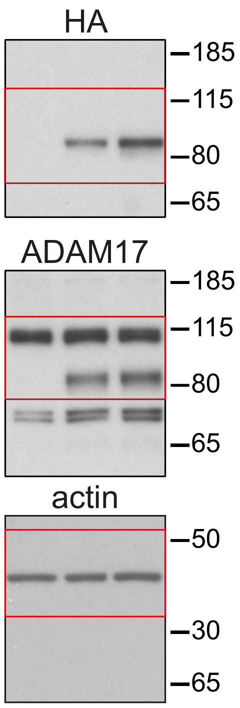

Fig. 5C

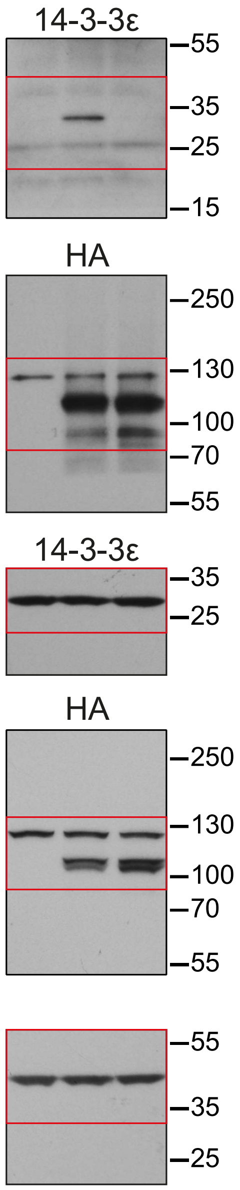

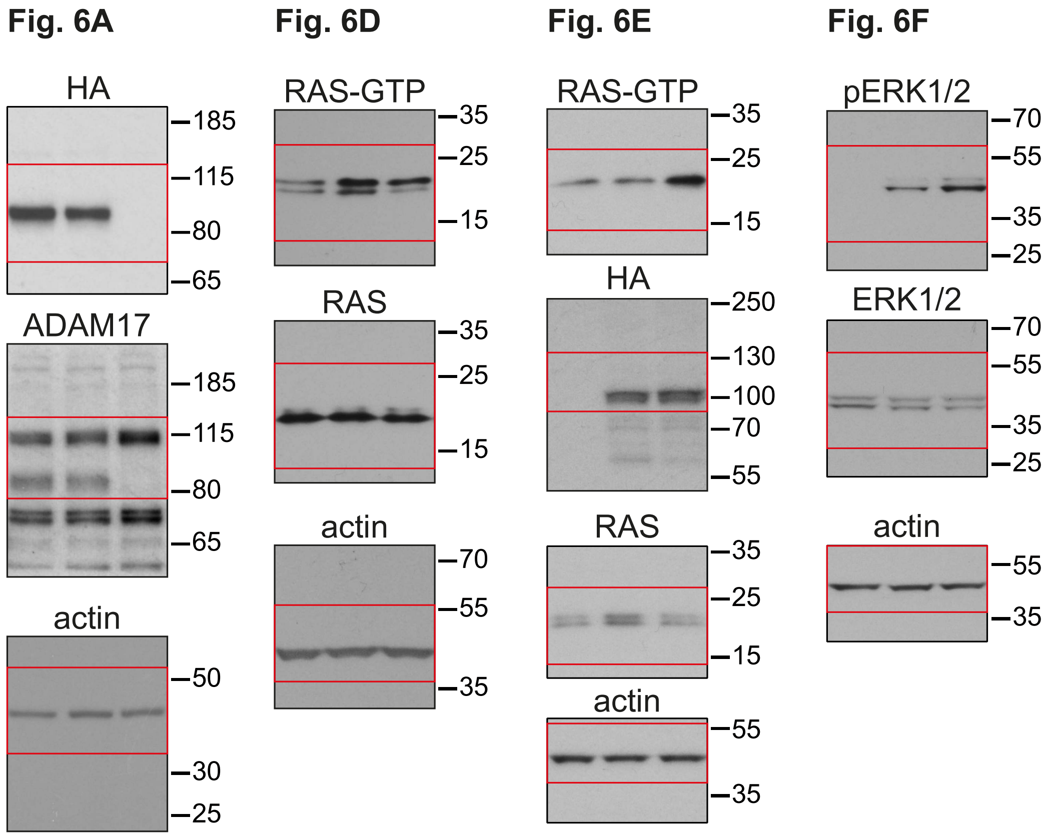

Journal of Cell Science • Supplementary information

Fig. S1A

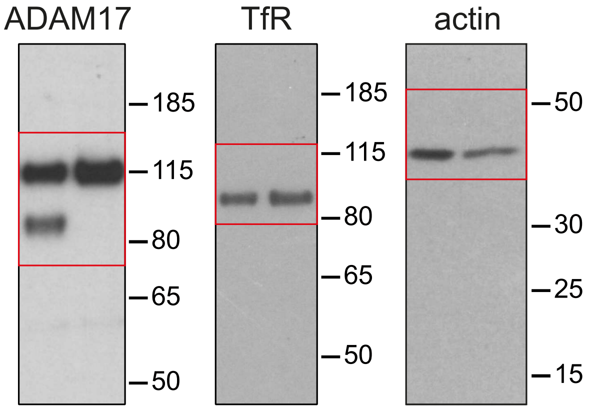

Fig. S2B

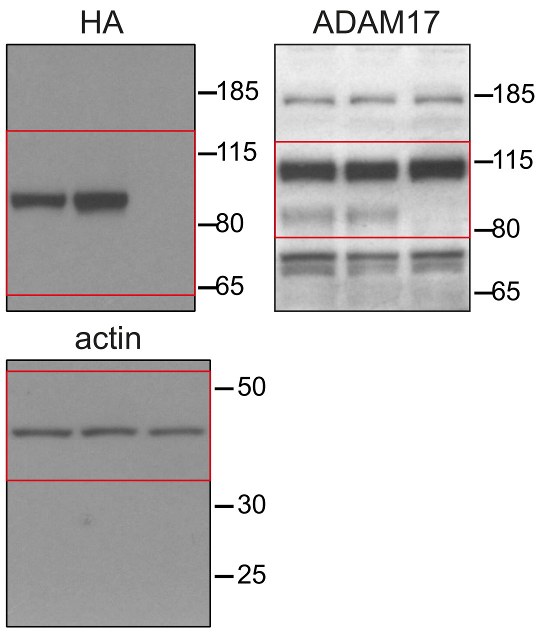

Fig. S2D

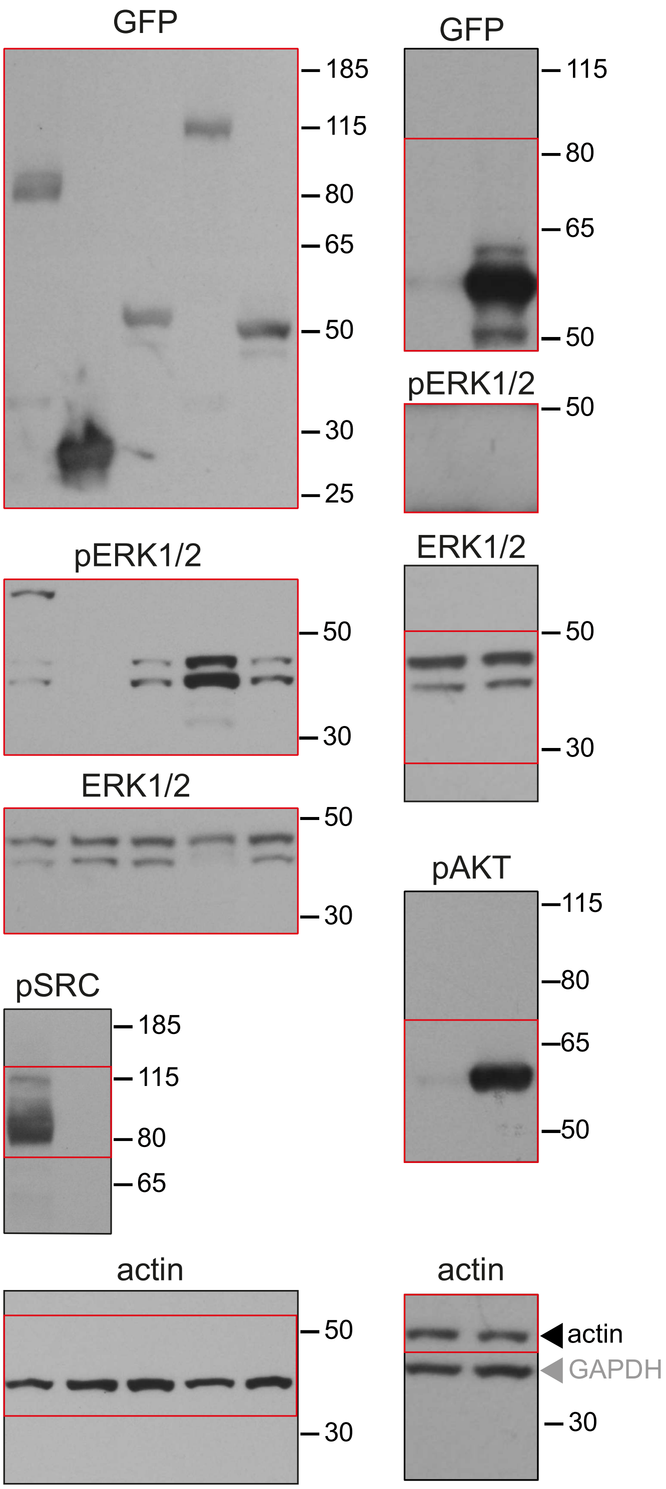

Journal of Cell Science • Supplementary information

J. Cell Sci.: doi:10.1242/jcs.259949: Supplementary information

Fig. S3B

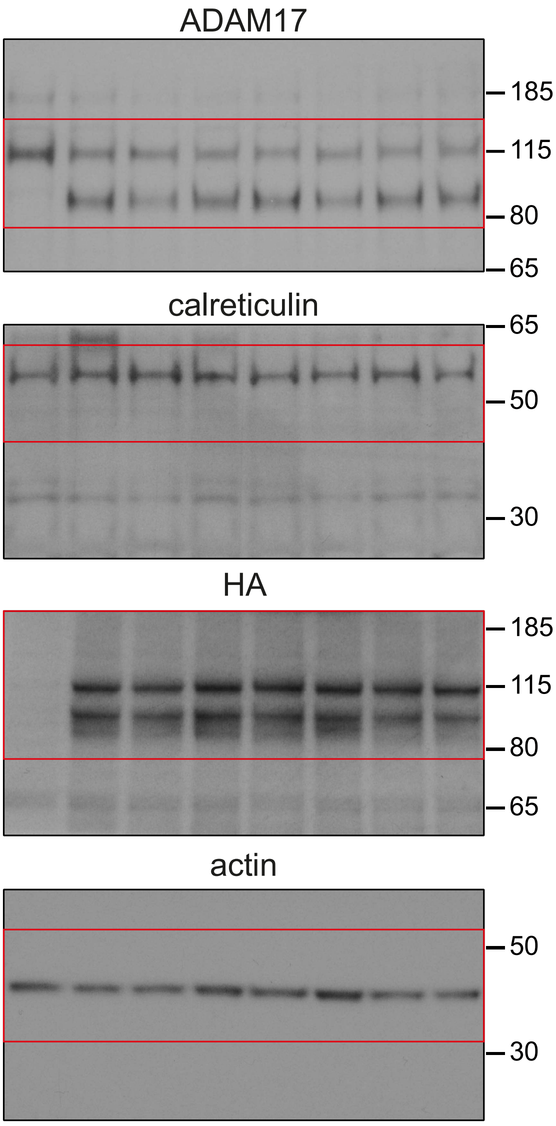

Fig. S4A

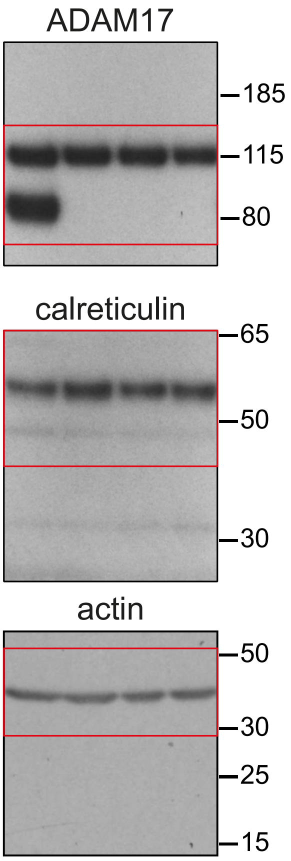

Journal of Cell Science • Supplementary information

Table S1. List of plasmids used in the study

Designation	Source or reference
pSpCas9n(BB)2A-Puro V2.0 (pX462 V2.0)	(Ran et al., 2013)
pHRSIN.pSFFV.blast-mouse iRhom2^{WT}-3xHA	This paper
pHRSIN.pSFFV.blast-mouse iRhom2-S58A-S60A-S83A-S85A-S87A-S357A-S359A-S360A-T361A (iRhom2$^{site1-3}$)-3xHA	This paper
pHRSIN.pSFFV.blast-human iRhom2^{WT}-3xHA	This paper
pHRSIN.pSFFV.blast-human iRhom2^{D188N}-3xHA	This paper
pHRSIN.pSFFV.blast-human iRhom2-S88A-S90A-S113A-S115A-S117A-S129A-S177A-Y229A-S323A-S325A-S328A-S385A-S387A-S388A-T389A(iRhom2^{pMUT})-3xHA	This paper
pLEX.puro-human iRhom2^{WT}-3xHA	(Künzel et al., 2018)
lentiviral packaging plasmid	(Adrain et al., 2012)
lentiviral envelope plasmid	(Adrain et al., 2012)
pEGFP-N1-EGFP (GFP)	Freeman lab
pEGFP-GFP-KRAS4A^{G12V}	(Grieve and Rabouille, 2014)
pEGFP-GFP-KRAS4A^{S17N}	This paper
pEGFP-GFP-KRAS4A^{G12S}	This paper
pEGFP-GFP-KRAS4A^{G12C}	This paper
pEGFP-GFP-KRAS4A^{G12D}	This paper
pEGFP-GFP-KRAS4B^{G12V}	This paper
pEGFP-GFP-SRC^{Y530F}	This paper
pEGFP-GFP-BRAF^{V600E}	This paper
pEGFP-GFP-HRAS^{G12V}	(Grieve and Rabouille, 2014)
pEGFP-AKT1^{E17K}	This paper
pLVX-TetOne-zeo	Michael van de Weijer
pLVX-TetOne-zeo-human iRhom2^{WT}-SNAP	This paper
pLVX-TetOne-zeo-human iRhom2^{I186T}-SNAP	This paper
pLVX-TetOne-zeo-human iRhom2^{D188Y}-SNAP	This paper
pLVX-TetOne-zeo-human iRhom2^{D188N}-SNAP	This paper
pLVX-TetOne-zeo-human iRhom2^{P189L}-SNAP	This paper
pLVX-TetOne-zeo-human iRhom2^{I186T,D188Y,P189L}-SNAP	This paper
pLVX-TetOne-zeo-human iRhom2^{I186T,D188N,P189L}-SNAP	This paper

Table S2. List of siRNAs used in the study

Name	Source	Catalogue number
Stealth RNAi siRNA negative control	Invitrogen	12935300
iRhom1 stealth siRNA	Thermo Fisher Scientific	HSS148857
iRhom2 stealth siRNA	Thermo Fisher Scientific	HSS128595

Table S3. List of primers used in the study

**Designation**	**Reference**	**Additional information**
gRNA targeting exon 3 of human *RHBDF1* (GGAACCATGAGTGAGGCCCC)	(Künzel et al., 2018)	gRNA targeting exon 3 of human *RHBDF1*
gRNA targeting exon 3 of human *RHBDF1* (GGGTGGCTTCTTGCGCTGCC)	(Künzel et al., 2018)	gRNA targeting exon 3 of human *RHBDF1*
gRNA targeting exon 10 of human *RHBDF1* (AGCCGTGTGCATCTATGGCC)	(Künzel et al., 2018)	gRNA targeting exon 10 of human *RHBDF1*
gRNA targeting exon 10 of human *RHBDF1* (CCGTCTCATGCTGCGAGAAC)	(Künzel et al., 2018)	gRNA targeting exon 10 of human *RHBDF1*
gRNA targeting exon 2 of human *RHBDF2* (GCAGAGCCGGAAGCCACCCC)	(Künzel et al., 2018)	gRNA targeting exon 2 of human *RHBDF2*
gRNA targeting exon 2 of human *RHBDF2* (GGGTCTCTTTCTCGGGTGGC)	(Künzel et al., 2018)	gRNA targeting exon 2 of human *RHBDF2*
gRNA targeting exon 9 of human *RHBDF2* (AAACTCGTCCATGTCATCATCACC)	(Künzel et al., 2018)	gRNA targeting exon 9 of human *RHBDF2*
gRNA targeting exon 9 of human *RHBDF2* (ACGGGTGCGATGCCATACGC)	(Künzel et al., 2018)	gRNA targeting exon 9 of human *RHBDF2*

Journal of Cell Science • Supplementary information

Table S4. List of cell lines used in the study

Designation	Source or reference	Additional information
HEK293T cells	Freeman lab	
HEK293T iRhom1/iRhom2 double-knockout (DKO)	(Künzel et al., 2018)	CRISPR/Cas9-mediated KO cell line
HEK239T iRhom1/iRhom2 DKO + $iRhom2^{WT}$	(Künzel et al., 2018)	HEK293T DKO cells transduced with pLEX.puro-human $iRhom2^{WT}$-3xHA
HEK293T iRhom1 knockout	This paper	CRISPR/Cas9-mediated KO cell line
HEK293T iRhom2 knockout	This paper	CRISPR/Cas9-mediated KO cell line
HEK293T iRhom1/iRhom2 DKO + $iRhom2^{WT}$	This paper	HEK293T DKO cells transduced with pHRSIN.pSFFV.blast-mouse $iRhom2^{WT}$-3xHA, used as control for HEK-DKO-$iRhom2^{site1-3}$
HEK239T iRhom1/iRhom2 DKO + $iRhom2^{site1-3}$	This paper	HEK293T DKO cells transduced with pHRSIN.pSFFV.blast-mouse $iRhom2^{site1-3}$-3xHA
HEK293T iRhom1/iRhom2 DKO + $iRhom2^{WT}$ (inducible)	This paper	HEK293T DKO cells transduced with pLVX-TetOne-zeo-human $iRhom2^{WT}$-SNAP, used as control for HEK DKO expressing iRhom2 TOC constructs
HEK293T iRhom1/iRhom2 DKO + $iRhom2^{I186T}$ (inducible)	This paper	HEK293T DKO cells transduced with pLVX-TetOne-zeo-human $iRhom2^{I186T}$-SNAP
HEK293T iRhom1/iRhom2 DKO + $iRhom2^{D188Y}$ (inducible)	This paper	HEK293T DKO cells transduced with pLVX-TetOne-zeo-human $iRhom2^{D188Y}$-SNAP
HEK293T iRhom1/iRhom2 DKO + $iRhom2^{D188N}$ (inducible)	This paper	HEK293T DKO cells transduced with pLVX-TetOne-zeo-human $iRhom2^{D188N}$-SNAP
HEK293T iRhom1/iRhom2 DKO + $iRhom2^{P189L}$ (inducible)	This paper	HEK293T DKO cells transduced with pLVX-TetOne-zeo-human $iRhom2^{P186L}$-SNAP
HEK293T iRhom1/iRhom2 DKO + $iRhom2^{I186T,D188Y,P189L}$ (inducible)	This paper	HEK293T DKO cells transduced with pLVX-TetOne-zeo-human $iRhom2^{I186T,D188Y,P189L}$-SNAP
HEK293T iRhom1/iRhom2 DKO + $iRhom2^{I186T,D188N,P189L}$ (inducible)	This paper	HEK293T DKO cells transduced with pLVX-TetOne-zeo-human $iRhom2^{I186T,D188N,P189L}$-SNAP
A549 cells*	(Bauer et al., 2018)	
A549 iRhom1/iRhom2 DKO	This paper	CRISPR/Cas9-mediated KO cell line
A549 iRhom1/iRhom2 DKO + $iRhom2^{WT}$	This paper	A549 DKO cells transduced at high multiplicity of infection (MOI) with pHRSIN.pSFFV.blast-human iRhom2WT-3xHA, used as control for A549-DKO-$iRhom2^{D188N}$
A549 iRhom1/iRhom2 DKO + $iRhom2^{D188N}$	This paper	A549 DKO cells transduced with pHRSIN.pSFFV.blast-human $iRhom2^{D188N}$-3xHA

Journal of Cell Science • Supplementary information

A549 iRhom1/iRhom2 DKO + iRhom2^{WT}	This paper	A549 DKO cells transduced at low MOI with pHRSIN.pSFFV.blast-human iRhom2^{WT}-3xHA, used as control for A549-DKO-iRhom2^{pMUT}
A549 iRhom1/iRhom2 DKO + iRhom2^{pMUT}	This paper	A549 DKO cells transduced with pHRSIN.pSFFV.blast-human iRhom2^{pMUT}-3xHA
A431 cells	Freeman lab	

*: tested negative for mycoplasma contamination

Table S5. List of antibodies used in the study

Name	Source	Catalogue number	Dilution
anti-ADAM17, rabbit polyclonal	Abcam	ab39162	1:2000
anti-beta-actin, mouse monoclonal	Santa Cruz	sc-47778	1:2000
anti-14-3-3 epsilon, rabbit polyclonal	CST	9635	1:500
anti-HA-HRP, rat monoclonal (clone 3F10)	Roche	12013819001	1:2000
anti-GFP, chicken polyclonal	Abcam	ab13970	1:4000
anti-phosphoERK1/2 (T202/Y204), rabbit monoclonal (clone 197G2)	CST	4377	1:500
anti-ERK1/2, rabbit polyclonal	CST	9102	1:1000
anti-V5, goat polyclonal	Santa Cruz	sc-83849	1:2000
anti-AKT,rabbit polyclonal	CST	9272	1:1000
anti-pSRC (Y416), rabbit polyclonal	CST	2101	1:1000
anti-pAKT (S473), rabbit polyclonal	CST	9271	1:1000
anti-calreticulin, rabbit polyclonal	Invitrogen	PA3-900	1:2000
anti-transferrin receptor1, mouse monoclonal (clone H68.4)	Invitrogen	13-6800	1:2000
anti-rabbit-HRP, goat polyclonal	CST	7074	1:2500
anti-goat-HRP, mouse monoclonal	Santa Cruz	sc2354	1:2500
anti-mouse-HRP, horse polyclonal	CST	7076	1:2500
anti-chicken-HRP, goat polyclonal	Novus Biologicals	NB7303	1:10000

Journal of Cell Science • Supplementary information
