# Supplementary material for: iRhom2 regulates ERBB signalling to promote KRAS-driven tumour growth of lung cancer cells
Source: J Cell Sci. 2022 Sep 8;135(17):jcs259949. doi: 10.1242/jcs.259949 (PMC9482348; doi:10.1242/jcs.259949)
Supplement: Reviewer comments [file joces-135-259949_review_history.pdf]

iRhom2 regulates ERBB signalling to promote KRAS-driven tumour growth of lung cancer cells

Boris Sieber, Fangfang Lu, Stephen M. Stribbling, Adam G. Grieve, Anderson J. Ryan and Matthew Freeman

DOI: 10.1242/jcs.259949

Editor: John Heath

Review timeline

Original submission:	22 February 2022
Editorial decision:	21 April 2022
First revision received:	14 July 2022
Accepted:	5 August 2022

Original submission

First decision letter

MS ID#: JOCES/2022/259949

MS TITLE: iRhom2 regulates ERBB signalling to promote KRAS-driven tumour growth of lung cancer cells

AUTHORS: Boris Sieber, Fangfang Lu, Stephen M Stribbling, Adam G Grieve, Anderson J Ryan, and Matthew Freeman
ARTICLE TYPE: Research Article

We have now reached a decision on the above manuscript.

To see the reviewers' reports and a copy of this decision letter, please go to: https://submit-jcs.biologists.org and click on the 'Manuscripts with Decisions' queue in the Author Area. (Corresponding author only has access to reviews.)

As you will see, the reviewers raise a number of criticisms that prevent me from accepting the paper at this stage. They suggest, however, that a revised version might prove acceptable, if you can address their concerns. If you think that you can deal satisfactorily with the criticisms on revision, I would be pleased to see a revised manuscript. We would then return it to the reviewers.

Please ensure that you clearly highlight all changes made in the revised manuscript. Please avoid using 'Tracked changes' in Word files as these are lost in PDF conversion.

I should be grateful if you would also provide a point-by-point response detailing how you have dealt with the points raised by the reviewers in the 'Response to Reviewers' box. Please attend to all of the reviewers' comments. If you do not agree with any of their criticisms or suggestions please explain clearly why this is so.

© 2022. Published by The Company of Biologists under the terms of the Creative Commons Attribution License (https://creativecommons.org/licenses/by/4.0/).

Reviewer 1

Advance summary and potential significance to field

ADAM17 is a transmembrane ectodomain sheddase of considerable interest because it plays a critical role in first line of defence in response to injury or infection by releasing EGF receptor ligands and TNF alpha, as well as shedding additional substrates. In the last 10 years, the authors have pioneered this area by showing that ADAM17 interacts with and is regulated by iRhom1 and iRhom2, a discovery that has been supported by the work of other groups. The present study is an advance because it extends this research area to cancer, showing that iRhom2/ADAM17 promotes the release of EGF family growth factors TGF alpha and amphiregulin, to enhance active mutant Ras signalling to activate iRhom2/ADAM17 activity via Erk-mediated phosphorylation of the iRhom2 cytoplasmic tail; hence a positive feedback loop. This model is of additional interest to cancer biologists, and has implications for future cancer therapy, because it builds on an emerging idea that active mutant Ras requires input from EGF receptors for full activation, and shows that growth factor release by ADAM17/iRhom2 is central to this process.

Comments for the author

This is an impressive study that begins using the HEK-293 cell line system into which the proteins of interest are transfected or knocked out, then extends to similar work in the A549 lung cancer cell line but using spheroid and in vivo cell growth assays. The use of naturally occurring iRhom2 mutations that are associated with cancer is an additional strength of the study.
I have the following points for the authors to address.
1. The experiments are in general expertly conducted, but some of the key data appear to be generated from a single CRISPR knockout clone of the cell line involved.
This is a problem because of potential clonal variation that could be due to intrinsic variation within the original cell line population or rare off-target effects of the knockout strategy. For example, in my lab we have recently generated triple knockout clones for a set of genes that we are interested in; two of the lines grow at a similar rate to wild-type, but one grows substantially slower. Our solution is to test multiple clones and/or reconstitute with the gene of interest or generate pools of knockouts rather than clones. It would be useful if the authors could address this limitation of their study with comments and additional supporting data with additional clones. This is particularly important for the spheroid and in vivo data in Figure 4, but also in all other figures that have used knockout HEK-293 and A549 clones.
2. The data are generally very impressive in terms of magnitudes of effects and obvious-looking statistical significance. However, I think that the statistics have been done incorrectly because they rely on t-tests. There are two problems with using t-tests. First, they should not be used on data with more than two groups; instead an ANOVA with multiple pairwise comparisons should be used. Second, they should only be used on data with a normal distribution, i.e. parametric. However the data in the manuscript is mostly non-parametric because it is in the form of percentage shedding or relative protein levels. To correctly do the stats, I would recommend first normalising the data by log transformation or arcsine of the square root, and then performing ANOVAs with multiple pairwise comparisons. It may be useful for the authors to check with a statistician to check that their statistics are done correctly.
3. Some of the western blotting data is in need of quantitation and statistical analysis to substantiate the interpretations made in the manuscript. This comment relates to Figure 2C and Figure 5A. On a related note, the nice quantitation in Figure S6 would seem more appropriate together with the data it is derived from in Figure 6D, and with the individual data points included on the bar charts, as was helpfully done for all other bar charts in the manuscript.
4. It is not clear why some of the shedding data is shown in two different forms. This was done for Figures 2A, 3B and 3C. If the authors can make no compelling case for showing some of the data in this way, I would suggest removing the extra dot plot panels.

Reviewer 2

Advance summary and potential significance to field

This a solid piece of work that advance our understanding on the role of iRhoms in oncogenic signaling.

© 2022. Published by The Company of Biologists under the terms of the Creative Commons Attribution License (https://creativecommons.org/licenses/by/4.0/).

Comments for the author

In this manuscript, Sieber, Lu et al investigate the role iRhoms in the growth of tumors driven by mutant KRas.
They show that overactive KRas phosphorytes iRhom; phosphorylated iRhom upregulates the activity of ADAM17, a protease that activates EGFR ligands. The subsequent activation of EGFR feeds forward the signals conveyed by KRas potentiating the deranged signaling pathway.

In general, this a solid piece of work that advance our understanding on the role of iRhoms in oncogenic signaling. The results shown are sound, properly controlled and support and, for the most part, support the conclusions made by the authors. I have to suggestions that may strengthen this report:

1. It has been amply shown that overexpression of oncogenic Ras, or that of other oncogenes, may induce a stable cell cycle arrest known as cellular senescence. This effect is particularly apparent in cells with wild type p53. The author should analyze cell proliferation and markers of cellular senescence, namely senescence-associated beta galactosidase activity after expression of activated oncogenes. If in the cells lines they use, oncogenic overexpression induces senescence, they should re-interpret their results accordingly.

2. The results in Figs. 4 and 5 clearly show that the knock-out of iRhoms has a much more pronounced effect than the impairment of phosphorylation. Accordingly, the effects on spheroid growth are also more evident in knock-out cells (compare Figs. 4B and 5D). Likely, the effect of mutations that prevent phosphorylation on tumor growth is even fainter. Overall, although the evidence shows that phosphorylation and interaction of 14-3-3epsylon, explains part of the effect of iRhoms on the shedding of Amphyregulin by ADAM17, likely iRhoms play additional roles. I would recommend the authors to include this possibility in the manuscript.

Minor points:
"We found that KRAS-induced shedding of TGFα was completely blocked in HEK293T double-knockout (DKO) cells mutant for both iRhom1 and iRhom2 (Fig. 1E, S1A)." In Fig. S1A the authors show that in DKO cells the processing of ADAM17 is disrupted. The authors should comment on this result fact or remove Fig. S1A.

First revision

<u>Author response to reviewers' comments</u>

Reviewer 1 – Comment 1. The experiments are in general expertly conducted, but some of the key data appear to be generated from a single CRISPR knockout clone of the cell line involved. This is a problem because of potential clonal variation that could be due to intrinsic variation within the original cell line population or rare off- target effects of the knockout strategy. For example, in my lab we have recently generated triple knockout clones for a set of genes that we are interested in; two of the lines grow at a similar rate to wild-type, but one grows substantially slower. Our solution is to test multiple clones and/or reconstitute with the gene of interest or generate pools of knockouts rather than clones. It would be useful if the authors could address this limitation of their study with comments and additional supporting data with additional clones. This is particularly important for the spheroid and in vivo data in Figure 4, but also in all other figures that have used knockout HEK-293 and A549 clones.

We recognise the importance of considering possible clonal variations using different approaches. As suggested, we produced additional A549 DKO clonal cell lines to complement the results of Figure 4. These three A549 DKO cell lines all show a drastic reduction of amphiregulin release (Fig. S4B), and a similar defect in spheroid growth (Fig. S4E). In addition, we have already reconstituted HEK293T iRhom1/2 DKO cells (Figs 2-3, S2-3) and A549 iRhom1/2 DKO cells (Figs 5-6, S5-6) with stably expressed iRhom2 constructs. Expression of iRhom2^{WT} rescued the ability of the cells to

© 2022. Published by The Company of Biologists under the terms of the Creative Commons Attribution License (https://creativecommons.org/licenses/by/4.0/).

release growth factor in presence of oncogenic KRAS and to efficiently form spheroids, thus confirming the specific contribution of iRhom activity in KRAS-induced growth factor release and spheroid formation. We have not used these additional lines to redo xenograft experiments for the practical reason that our collaborator with xenograft experience has now retired and closed his lab. Nevertheless, we hope that editors agree that the point is sufficiently addressed with the shedding and spheroid experiments.

Reviewer 1 – Comment 2. The data are generally very impressive in terms of magnitudes of effects and obvious-looking statistical significance. However, I think that the statistics have been done incorrectly because they rely on t-tests. There are two problems with using t-tests. First, they should not be used on data with more than two groups; instead an ANOVA with multiple pairwise comparisons should be used. Second, they should only be used on data with a normal distribution, i.e. parametric. However, the data in the manuscript is mostly non-parametric because it is in the form of percentage shedding or relative protein levels. To correctly do the stats, I would recommend first normalising the data by log transformation or arcsine of the square root, and then performing ANOVAs with multiple pairwise comparisons. It may be useful for the authors to check with a statistician to check that their statistics are done correctly.

As suggested, we asked Dr Nicolas Guex and Dr. Maxime Jan of the Bioinformatics Competence Center (University of Lausanne, Switzerland) to perform the statistical analysis of the data: The required assumptions for one-way ANOVA were tested for each statistical test. Bartlett's test was used to evaluate the variance homoscedasticity and Shapiro-Wilk test was used to verify residual normality. A log10 transformation was applied when heteroscedasticity or non-normal distribution of residuals was detected. Pairwise comparison was performed using a post-hoc Tukey's test. The manuscript and figures have been updated with the ANOVA post hoc p-values.

Reviewer 1 – Comment 3. Some of the western blotting data is in need of quantitation and statistical analysis to substantiate the interpretations made in the manuscript.
This comment relates to Figure 2C and Figure 5A. On a related note, the nice quantitation in Figure S6 would seem more appropriate together with the data it is derived from in Figure 6D, and with the individual data points included on the bar charts, as was helpfully done for all other bar charts in the manuscript.

Thank you for pointing this out, we have added the quantification of 14-3-3 level in Figure 2C as Figure S2C, and the quantification of iRhom2 level in Figure 5A is shown. We also agree in adding the quantifications of Figure 6D-E directly below the corresponding western blots.

Reviewer 1 – Comment 4. It is not clear why some of the shedding data is shown in two different forms. This was done for Figures 2A, 3B and 3C. If the authors can make no compelling case for showing some of the data in this way, I would suggest removing the extra dot plot panels.

This is a fair point and we have removed the graphs of the percentage of induction from Figure 2A, 3B and 3C, as the graphs of the substrate release are already sufficient for the interpretation.

Reviewer 2 – Comment 1. It has been amply shown that overexpression of oncogenic Ras, or that of other oncogenes, may induce a stable cell cycle arrest known as cellular senescence. This effect is particularly apparent in cells with wild type p53.
The author should analyze cell proliferation and markers of cellular senescence, namely senescence-associated beta galactosidase activity after expression of activated oncogenes. If in the cells lines they use, oncogenic overexpression induces senescence, they should re-interpret their results accordingly.

We agree that, in addition to their ability to induce growth factor shedding, the ability of the oncogenes to induce cellular senescence in HEK293T should be assessed. To do so, we transiently transfected 100 ng of oncogene per well of a 24-well plate, as we did for the AP- shedding assay, and we measured senescence-associated ß-galactosidase activity (Cell Signaling Technology, #9860) 24 hrs after transfection. The level of senescence-associated ß-galactosidase is similar to that in control (GFP) upon expression of oncogenic KRAS, HRAS, BRAF, This demonstrates that senescence

© 2022. Published by The Company of Biologists under the terms of the Creative Commons Attribution License (https://creativecommons.org/licenses/by/4.0/).

is negligible in the conditions in which we performed the AP-shedding assays. It is consistent with the earlier studies of oncogene- induced senescence, which is a slower process, usually measured four days after oncogene expression (Innes and Gil, 2019; Serrano et al., 1997).

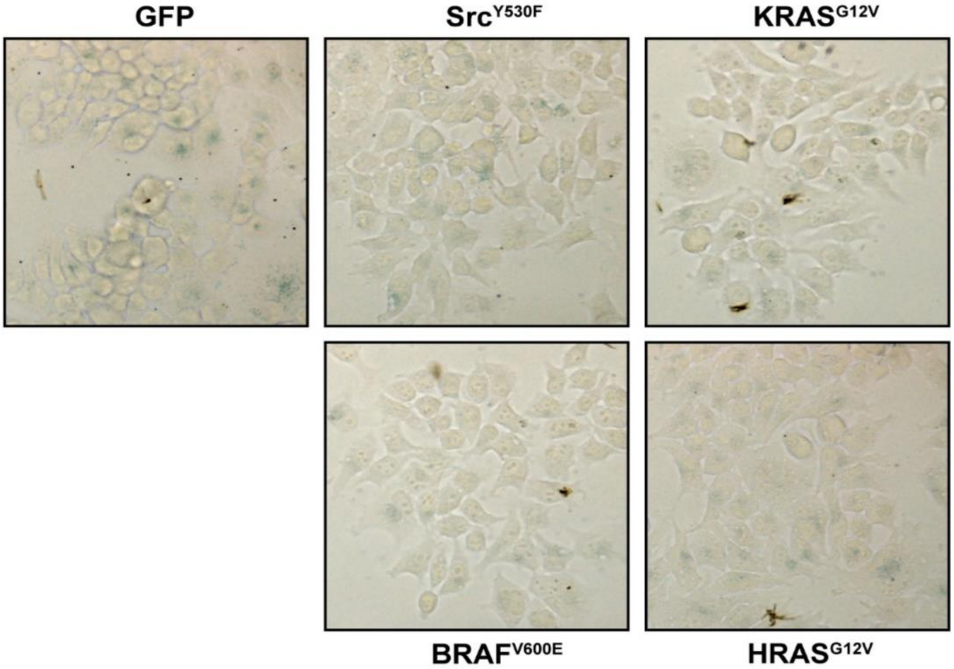

Senescence-associated β-galactosidase staining upon transient expression of oncogenes. HEK293T cells seeded in 24-well plate were transfected with 100 ng of the indicated oncogene, or GFP as a control, for 24 hrs before assessing the level of β- galactosidase activity using the Cell Signaling Technology kit (#9860) according to the manufacturer's instructions.

Innes, A. J., and Gil, J. (2019). IMR90 ER:RAS: A Cell Model of Oncogene-Induced Senescence. Methods Mol Biol *1896*, 83-92.

Serrano, M., Lin, A. W., McCurrach, M. E., Beach, D., and Lowe, S. W. (1997). Oncogenic ras provokes premature cell senescence associated with accumulation of p53 and p16INK4a. Cell *88*, 593-602.

Reviewer 2 – Comment 2. The results in Figs. 4 and 5 clearly show that the knock-out of iRhoms has a much more pronounced effect than the impairment of phosphorylation. Accordingly, the effects on spheroid growth are also more evident in knock-out cells (compare Figs. 4B and 5D). Likely, the effect of mutations that prevent phosphorylation on tumor growth is even fainter. Overall, although the evidence shows that phosphorylation, and interaction of 14-3-3epsylon, explains part of the effect of iRhoms on the shedding of Amphyregulin by ADAM17, likely iRhoms play additional roles. I would recommend the authors to include this possibility in the manuscript.

We agree that clarification about the contributions of iRhoms in KRAS-induced tumorigenesis is needed. As we have demonstrated in our previous works, iRhoms plays two critical roles in the control of ADAM17. First, iRhoms are required for the trafficking of ADAM17 from the ER to the Golgi, where the sheddase undergoes maturation by pro- protein convertase such as furins. The loss of ADAM17 maturation therefore confirms the absence of iRhom activity in DKO cell lines. Second, the post-translational modifications of iRhoms at the plasma membrane induces the activation of ADAM17 at the plasma membrane.

In Figure 5, we have focused on this second role of iRhoms at the plasma membrane and, as noted, our data demonstrate the requirement of iRhom2 phosphorylation for effective growth factor release and spheroid growth. We did not expect iRhom2^{pMUT} to fully phenocopy A549-DKO cells, as the former still present basal ADAM17 activity. As suggested, phosphorylation-independent functions of iRhoms, such as recruitment of other interactors, may have also contributed to the stronger phenotype observed in Figure 4.

© 2022. Published by The Company of Biologists under the terms of the Creative Commons Attribution License (https://creativecommons.org/licenses/by/4.0/).

We have modified the manuscript to clarify our focus on the second function of iRhoms on ADAM17 activity, and to include the possibility of additional roles play by iRhoms in lung cancer cells.

Reviewer 2 – Minor comment. “We found that KRAS-induced shedding of TGFa was completely blocked in HEK293T double-knockout (DKO) cells mutant for both iRhom1 and iRhom2 (Fig. 1E, S1A).” In Fig. S1A the authors show that in DKO cells the processing of ADAM17 is disrupted. The authors should comment on this result fact or remove Fig. S1A.
See response to previous comment.

Second decision letter

MS ID#: JOCES/2022/259949

MS TITLE: iRhom2 regulates ERBB signalling to promote KRAS-driven tumour growth of lung cancer cells

AUTHORS: Boris Sieber, Fangfang Lu, Stephen M Stribbling, Adam G Grieve, Anderson J Ryan, and Matthew Freeman
ARTICLE TYPE: Research Article

I am happy to tell you that your manuscript has been accepted for publication in Journal of Cell Science, pending standard ethics checks.

Reviewer 1

Advance summary and potential significance to field

ADAM17 is a transmembrane ectodomain sheddase of considerable interest because it plays a critical role in first line of defence in response to injury or infection by releasing EGF receptor ligands and TNF alpha, as well as shedding additional substrates. In the last 10 years, the authors have pioneered this area by showing that ADAM17 interacts with and is regulated by iRhom1 and iRhom2, a discovery that has been supported by the work of other groups. The present study is an advance because it extends this research area to cancer showing that iRhom2/ADAM17 promotes the release of EGF family growth factors TGF alpha and amphiregulin, to enhance active mutant Ras signalling to activate iRhom2/ADAM17 activity via Erk-mediated phosphorylation of the iRhom2 cytoplasmic tail; hence a positive feedback loop. This model is of additional interest to cancer biologists, and has implications for future cancer therapy because it builds on an emerging idea that active mutant Ras requires input from EGF receptors for full activation, and shows that growth factor release by ADAM17/iRhom2 is central to this process.

Comments for the author

I am happy that the authors have taken on board my comments and applied them to the revised version.

Reviewer 2

Advance summary and potential significance to field

This a solid piece of work that advances our understanding on the role of iRhoms in oncogenic signaling.

Comments for the author

The authors have satisfactorily addressed the concerns on the previous version of the manuscript.

© 2022. Published by The Company of Biologists under the terms of the Creative Commons Attribution License (https://creativecommons.org/licenses/by/4.0/).
